# Kaposi's Sarcoma Herpesvirus microRNAs Target Caspase 3 and Regulate Apoptosis

**DOI:** 10.1371/journal.ppat.1002405

**Published:** 2011-12-08

**Authors:** Guillaume Suffert, Georg Malterer, Jean Hausser, Johanna Viiliäinen, Aurélie Fender, Maud Contrant, Tomi Ivacevic, Vladimir Benes, Frédéric Gros, Olivier Voinnet, Mihaela Zavolan, Päivi M. Ojala, Juergen G. Haas, Sébastien Pfeffer

**Affiliations:** 1 Architecture et Réactivité de l'ARN, Institut de Biologie Moléculaire et Cellulaire du CNRS, Université de Strasbourg, Strasbourg, France; 2 Max von Pettenkofer-Institute, Ludwig-Maximilians-University Munich, Munich, Germany; 3 Biozentrum der Universität Basel and Swiss Institute of Bioinformatics, Basel, Switzerland; 4 Genome-Scale Biology Program, Biomedicum Helsinki and Institute of Biomedicine, University of Helsinki, Helsinki, Finland; 5 GeneCore (Genomics Core Facility), EMBL, Heidelberg, Germany; 6 Immunologie et Chimie Thérapeutiques UPR 9021, Institut de Biologie Moléculaire et Cellulaire du CNRS, Université de Strasbourg, Strasbourg, France; 7 Institut de Biologie Moléculaire des Plantes du CNRS, Strasbourg, France; 8 Foundation for the Finnish Cancer Institute, Helsinki, Finland; 9 Division of Pathway Medicine, University of Edinburgh Medical School, Edinburgh, United Kingdom; Oregon Health & Science University, United States of America

## Abstract

Kaposi's sarcoma herpesvirus (KSHV) encodes a cluster of twelve micro (mi)RNAs, which are abundantly expressed during both latent and lytic infection. Previous studies reported that KSHV is able to inhibit apoptosis during latent infection; we thus tested the involvement of viral miRNAs in this process. We found that both HEK293 epithelial cells and DG75 cells stably expressing KSHV miRNAs were protected from apoptosis. Potential cellular targets that were significantly down-regulated upon KSHV miRNAs expression were identified by microarray profiling. Among them, we validated by luciferase reporter assays, quantitative PCR and western blotting caspase 3 (Casp3), a critical factor for the control of apoptosis. Using site-directed mutagenesis, we found that three KSHV miRNAs, miR-K12-1, 3 and 4-3p, were responsible for the targeting of Casp3. Specific inhibition of these miRNAs in KSHV-infected cells resulted in increased expression levels of endogenous Casp3 and enhanced apoptosis. Altogether, our results suggest that KSHV miRNAs directly participate in the previously reported inhibition of apoptosis by the virus, and are thus likely to play a role in KSHV-induced oncogenesis.

## Author Summary

MiRNAs are small, non-coding RNAs that regulate gene expression post-transcriptionally via binding to complementary sites in target mRNAs. This evolutionary conserved regulatory system is present in most eukaryotes, and it has recently been shown that certain viruses have evolved to express their own miRNAs. Due to their non-immunogenic nature, viral miRNAs represent an efficient tool for the virus to control its environment. Here we show that KSHV miRNAs are involved in the control of apoptosis both when expressed in stable cell lines and in the context of viral infection. Using a microarray based approach we identified putative cellular targets, among which the effector caspase 3 is targeted by three of the viral miRNAs. Finally, we showed that blocking these miRNAs in infected cells resulted both in increased Casp3 levels and a higher apoptosis rate. These findings indicate that miRNAs of viral origin are key players in cell death inhibition by KSHV.

## Introduction

The development of cancer is linked to six major hallmarks that explain how cells transgress from a normal to a neoplastic state, including (i) sustained proliferative signaling, (ii) evasion of growth suppression, (iii) activated invasion and metastasis, (iv) enabled replicative immortality, (v) induced angiogenesis and (vi) resistance to cell death [1]. There is ample evidence that programmed cell death or apoptosis functions as a barrier to cancer development (reviewed in [2]). Many different factors, including environmental ones, contribute to the origin and progression of cancer. For example, infection by microbial pathogens sometimes leads to tumor development. Several viruses have been recognized as causal agents of specific types of cancer, and up to 20% of all human cancers are associated with single or multiple viral infections. One such oncogenic virus is Kaposi's sarcoma-associated herpesvirus (KSHV), the primary etiological agent of Kaposi's sarcoma, which is a highly angiogenic tumor most probably arising from the endothelium and developing primarily in immunocompromised individuals. KSHV-infection is also associated with aggressive lymphomas such as primary effusion lymphoma and multicentric Castleman's disease [3]. Like many viruses, KSHV has been shown to inhibit apoptosis, and possesses a truly impressive arsenal to do so (reviewed in [4], [5]).

Viruses have acquired an extraordinary capacity to evolve and adapt to their host, which translates into an incessant battle between the infected organism and the virus. One of the latest discoveries reflecting this continuous arms race is that certain mammalian viruses encode for miRNAs. In mammals, miRNAs constitute one of the most important classes of regulatory RNAs [6], [7]. Their biogenesis involves the processing of a large primary transcript into a stem-loop pre-miRNA, ultimately leading to the mature single stranded ∼22 nt miRNA (reviewed in [7]–[11]). This functional miRNA is incorporated into an RNA-induced silencing complex (RISC) that invariably contains a member of the Argonaute protein family. Once loaded, the active RISC can be directed towards its messenger RNA target to regulate, predominantly negatively, its translation (see references [12], [13] for review). The fact that target RNAs are frequently destabilized justifies the use of large-scale approaches to look at global changes in transcriptomic profiles as a way to identify miRNA targets [14]. To date, the vast majority of reported miRNA/mRNA interactions involve binding of the miRNA to the 3′ untranslated region (UTR) of the transcript through an imperfect base-pairing mechanism in which nucleotides 2 to 8 of the miRNA (the seed) appear to play an important role [15]. However, other types of interactions, such as binding in the coding sequence or in the 5′ UTR, or with bulges in the seed region, have also been reported [16]–[18].

The use of small non-coding RNAs such as miRNAs to regulate gene expression makes perfect sense for viruses, allowing them to modulate the cellular environment in a non-immunogenic manner [19]. The first virus-encoded miRNAs were identified in Epstein-Barr virus [20], and subsequent studies concluded that many herpesviruses, including Kaposi's sarcoma herpesvirus (KSHV) encode miRNAs (reviewed in [21]). KSHV has been shown to encode 12 miRNAs [22]–[25], which are clustered in the vicinity of the major KSHV latency transcript, K12. KSHV-miR-K12-1 to miR-K12-9, and miR-K12-11 are located in the intron of the larger kaposin transcript, while miR-K12-10 maps to the coding region, and miR-K12-12 resides within the 3′ UTR of the K12 coding sequence. Some cellular targets of KSHV miRNAs have been identified, mostly for miR-K12-11, which shares an identical seed sequence with the cellular miRNA miR-155 [26], [27].

Here, we show that KSHV miRNAs also contribute to the inhibition of apoptosis in infected cells. We show that cell lines expressing KSHV miRNAs are less sensitive to both caspase-dependent and -independent apoptosis induction by staurosporine or etoposide. Using a microarray approach, we identified caspase 3 (Casp3) as a target of some of these viral miRNAs. Casp3 is a well-known effector caspase (reviewed in [28]) that is critical for apoptosis induction. Using site-directed mutagenesis, we found that KSHV miR-K12-1, K12-3 and K12-4-3p are responsible for Casp3 regulation. Finally, by blocking the function of these miRNAs in infected cells, we showed that both Casp3 levels and apoptosis were increased.

## Results

### Cell lines expressing KSHV miRNAs are less sensitive to apoptosis

**Figure 1:**
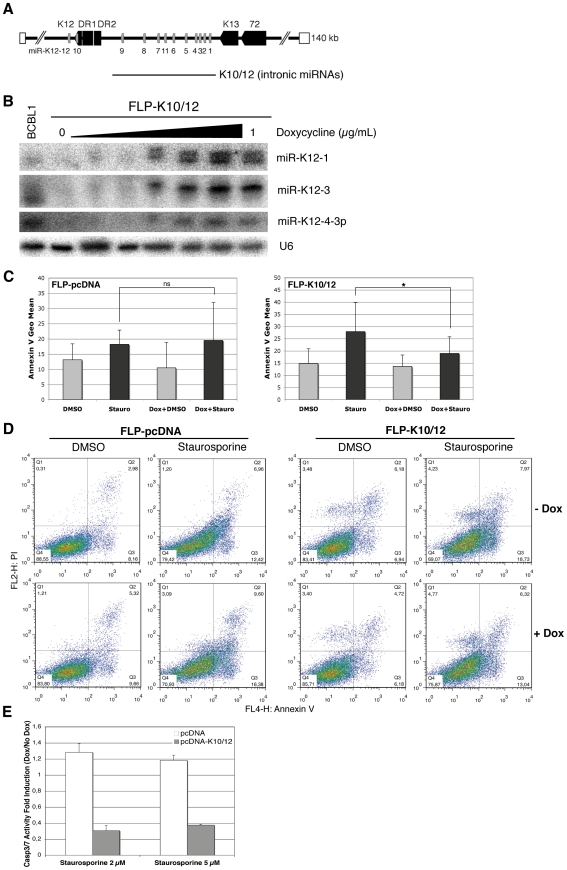
HEK293 cells expressing KSHV miRNAs are less sensitive to apoptosis. **A.** Schematic representation of KSHV miRNA genomic localization, and of the K10/12 construct that was used for their expression. **B.** Northern blot analysis of inducible FLP-K10/12 cells. Cells were grown for 48h with increasing concentration of doxycycline (0 to 1 µg/mL); a concentration of 1 µg/mL was used in the following experiments. KSHV latently infected BCBL-1 cells were used as a positive control. **C.** Statistical analysis of apoptosis induction measured by Annexin V binding assay in FLP-pcDNA control cell line (left panel) or FLP-K10/12 (right panel) grown continuously in doxycycline-containing medium, and treated with DMSO or staurosporine for 8 h. Error bars represent the standard deviation observed for 6 biological replicates; a significant difference (p = 0.0306) of apoptosis induction is observed between the non-treated and doxycycline-treated K10/12 expressing cell lines, but not for the pcDNA cell line. **D.** Dot plot examples of a representative FACS analysis of annexin V and propidium iodide (PI) levels in FLP-pcDNA (left panel) or FLP-K10/12 cells (right panel). **E.** The same cells treated for 8 h with DMSO or 2 and 5 µM staurosporine, were assayed for Casp3/7 activity after addition of a luminescent substrate for the caspases, and normalized to the total protein content. The ratio between doxycycline-treated and non-treated cells is given.

We generated inducible HEK293 cells (FLP-293) expressing the intronic KSHV miRNAs under a doxycycline-inducible CMV promoter. To this end, the sequence spanning the ten intronic miRNAs miR-K12-1 to 9 and miR-K12-11 (K10/12) (Figure 1A) was inserted into the pcDNA5/FRT/TO plasmid, and used to transfect Flp-In T-Rex-293 cells. Stable cell lines were obtained by hygromycin selection, and subsequently named FLP-K10/12. As a negative control, we generated stable cells transfected with a pcDNA5 plasmid with no insert, that we then named FLP-pcDNA. We verified by northern blot analysis that doxycycline treatment readily induced the expression of the miRNAs to level similar to that found in the KSHV-infected BCBL-1 cells [29] (Figure 1B). In all following experiments, we used a final concentration of doxycycline of 1 µg/mL. We also measured by northern blot analysis the level of KSHV miRNAs expression in the induced FLP-K10/12 cells and compared it to KSHV-infected BCBL-1 and BC3 cells [30]. We found that expression of the miRNAs was slightly higher than in BCBL-1, but lower than in BC-3 cells (Figure S1), suggesting expression close to physiological levels. To assess the effect of KSHV miRNA expression on apoptosis, we first grew the FLP-pcDNA and -K10/12 cell lines in the presence of doxycycline to induce expression of the viral miRNAs, and then treated them for 8 h with 2 µM of staurosporine, a well-described inducer of apoptosis [31], or DMSO as a control. To measure the effect of this treatment on apoptosis we used Annexin V binding assay, which allows quantification of the level of phosphatidylserine exposure at the outer membrane side, a well characterized event of early apoptosis [32]. In addition, cells were labeled with propidium iodide (PI), staining both apoptotic and necrotic cells. Statistical analysis of six independent cell-sorting experiments revealed that Annexin V binding levels following staurosporine treatment were not significantly different in the presence or absence of doxycycline for the control FLP cell line (Figure 1C). In contrast, concerning the FLP-K10/12 cell line, a statistically significant decrease in Annexin V levels after staurosporine treatment was observed following doxycycline-induced expression of the microRNAs (Figure 1C). Figure 1D shows one representative experiment of the six biological replicates. In order to get an independent measure of apoptosis, we monitored the activity of effector caspases using a DEVD-aminoluciferin substrate for Casp3 and Casp7 that is measurable by a luciferase assay. As shown in Figure 1E, the luminescent Casp3/7 activity induced by 2 or 5 µM of staurosporine treatment of the stable FLP-K10/12 cells was sharply decreased (2.5 to 3 times) upon doxycycline induction of the KSHV miRNAs expression, while it remained unchanged in the control FLP-pcDNA cells.

**Figure 2:**
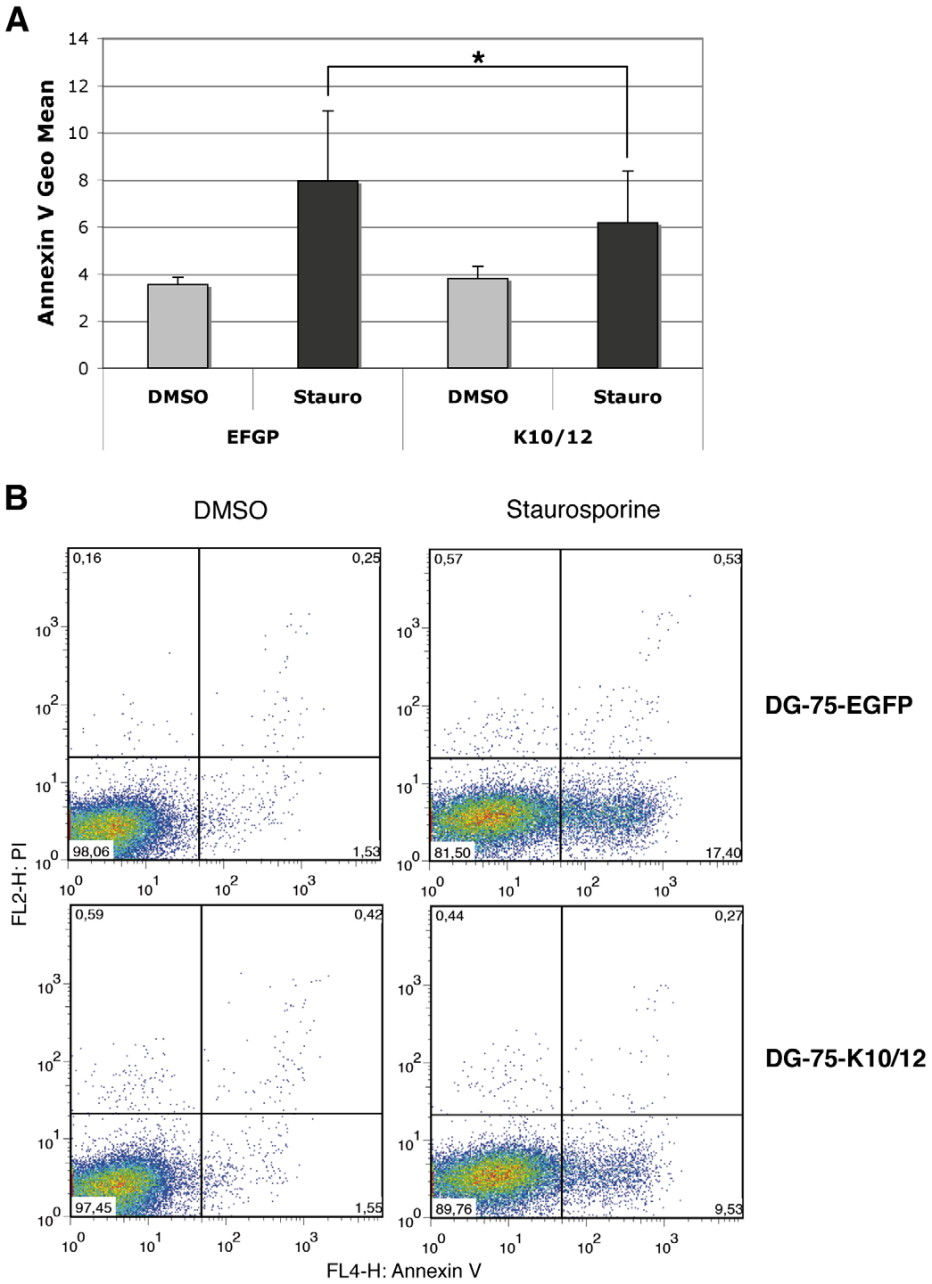
DG-75 cells expressing KSHV miRNAs are less sensitive to apoptosis. **A.** Statistical analysis of apoptosis induction measured by Annexin V binding assay in stable DG-75-EGFP cells as a control or DG-75-K10/12, treated for 8 h with DMSO or staurosporine. Error bars represent the standard deviation observed for 4 biological replicates; a significant difference of apoptosis induction (p = 0.0355) is observed between the EGFP and the K10/12 expressing cell lines. **B.** Dot plot examples of a representative FACS analysis of annexin V and propidium iodide (PI) levels in EGFP (upper panels), or K10/12 DG-75 cells (lower panels).

To monitor the effect of KSHV miRNAs on apoptosis in a cell line more physiologically relevant for KSHV infection, we used the previously described DG-75-K10/12 cells -a Burkitt lymphoma cell line [33], [34] lentivirally transduced with a construct expressing KSHV intronic miRNAs [35]-, and measured the effect of KSHV miRNAs expression on apoptosis in either the DG-75-K10/12 cells or the DG-75-EGFP control cells. Statistical analysis of four independent experiments confirmed that staurosporine treatment readily induced phosphatidylserine exposure in the control cell line, but that this induction was significantly reduced in the K10/12-expressing cells (Figure 2A). A representative experiment of the biological replicates (Figure 2B) shows that the percentage of Annexin V positive cells dropped almost two-fold in DG-75-K10/12 cells *vs*. DG-75-EGFP cells after 8 h of staurosporine treatment. As opposed to the FLP-293 cells, we were unable to induce Casp3/7 activity with staurosporine in the DG75 cells (data not shown). In line with this observation, it has been reported previously that in this particular cell line, the apoptotic protease-activating factor 1 (APAF-1) was sequestered at the plasma membrane, which prevents caspase activation [36].

### Microarray analysis of KSHV miRNAs-expressing cell lines

In order to identify putative cellular targets of KSHV miRNAs involved in the miRNA-induced anti-apoptotic phenotype, we used a microarray approach on the two main cell types that are infected *in vivo* by KSHV: endothelial cells and B lymphocytes. In addition to the already described DG-75-K10/12 and DG-75-EGFP cells, we also generated by lentiviral transduction endothelial cells EA.hy926 [37] expressing the K10/12 construct or EGFP as a control. In order to determine the relative expression of KSHV miRNAs were expressed in the DG-75-K10/12 cells, we cloned the small RNA population of these cells and analyzed it by Solexa deep-sequencing. As can be seen in Table S1, KSHV miRNAs represented more than 18% of the total miRNAs in this cell line, which is slightly less than what has been previously described for BCBL1 cells ([38] and data not shown). All intronic miRNAs accumulated to measurable levels with the exception of miR-K12-11, which seemed to be expressed at a low level. We then measured by qRT-PCR the levels of some KSHV miRNAs expression in EA.hy926 and DG-75-K10/12 cells compared to BCBL-1 cells (Table S2). The levels of viral miRNAs expression in both cell lines correlated very well (r = 0,93) (Figure S2).

DG-75 and EA.hy926 EGFP control- and miRNA- expressing cell lines were analyzed in triplicate on Affymetrix Human Genome U133 Plus 2.0 microarrays. The clustering of the gene expression profiles primarily correlated with the cell line (DG-75 *vs.* EA.hy926), but also within each cell line with the expression of KSHV miRNAs (Figure S3). In addition, the changes in gene expression levels following the KSHV miRNAs transduction correlated weakly (r = 0.19) but significantly (p<10^−15^ at Pearson's test) between the two cell lines (Figure S4).

**Figure 3:**
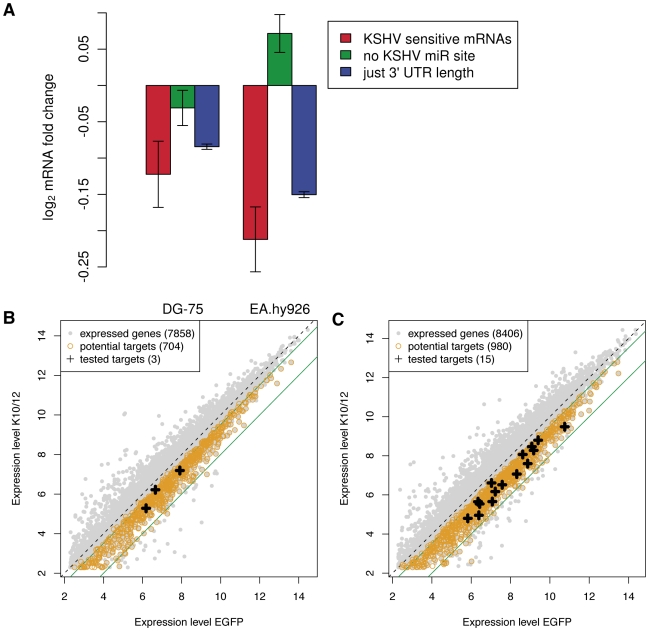
Microarray analysis of KSHV miRNAs expressing cell lines. **A.** Changes in expression levels of KSHV miRNA sensitive mRNAs, mRNAs without KSHV miRNA seed matches in their 3′ UTR, and randomized sets of genes with the same 3′ UTR length distribution as KSHV miRNA sensitive mRNAs. The analysis was performed separately in DG-75 and EA.hy926 cells. Error bars represent 95% confidence interval on the mean fold change in gene expression upon transducing the KSHV miRNAs. Dot plot representation of the changes in gene expression observed in the DG-75 cells (**B**), and in the Ea.hy926 cells (**C**). The potential targets (black crosses) tested by luciferase assay were selected among the genes down-regulated 1.4 to 4 fold (indicated by the green lines).

Target recognition by miRNAs involves a number of determinants, the most important of which appears to be perfect base-pairing of nucleotides 2–7 of the miRNA (the seed), together with either an adenosine opposite miRNA nucleotide 1, or an additional base pair involving the 8^th^ nucleotide of the miRNA [15]. In single miRNA transfection experiments one typically observes that the mRNAs that carry matches to the transfected miRNA are significantly down-regulated in response to transfection compared to mRNAs that do not carry such matches. To determine whether the KSHV miRNAs significantly influenced gene expression levels in a complex experiment such as ours, in which multiple miRNAs are simultaneously induced, we designed the following test. We first computed a KSHV miRNA sensitivity score for each mRNA, defined as the sum over all KSHV miRNAs, the number of matches of the 3′ UTR to the seed of the KSHV miRNA multiplied by the relative abundance of the KSHV miRNA. The relative abundances of the KSHV miRNAs were determined using the DG-75-K10/12 small RNAs deep-sequencing data. The KSHV miRNA sensitivity scores are reported in Dataset S1. We then compared the change in expression level of the 1000 mRNAs with highest KSHV miRNA sensitivity score and of mRNAs with no seed matches to the KSHV miRNAs in the 3′ UTR and found that the KSHV miRNA sensitive mRNAs were significantly down-regulated in both KSHV miRNA expressing DG-75 and EA.hy926 cells (p<10^−3^ and p<10^−15^, respectively in Wilcoxon's rank sum test). We observed however, that the 3′ UTRs of the 1000 mRNAs with highest KSHV sensitivity were on average ten times longer than the 3′ UTRs with no seed matches (Figure S5). To test whether differences in 3′ UTR length alone could account for the down-regulation of the KSHV sensitive mRNAs, we computed the average fold change of 1000 mRNAs sampled in such way that their 3′ UTR length distribution was the same as that of the KSHV sensitive mRNAs (Figure 3A, blue bars). We repeated this procedure 1000 times and found that the set of 1000 KSHV sensitive mRNAs still exhibited a stronger down-regulation compared to mRNAs of similar 3′ UTR length (Figure 3A, red bars) (p = 0.036 and 0.002, respectively for the expression changes computed from the DG-75 and EA.hy926 samples). Therefore, the 3′ UTR length alone cannot explain the magnitude of down-regulation of the most KSHV sensitive mRNAs in response to KSHV miRNA expression. These results indicated that KSHV miRNAs exert a detectable effect on mRNA expression in these cell lines and motivated us to proceed with further characterization of candidate direct targets.

As KSHV putative direct targets we extracted transcripts that were significantly down-regulated significantly in the replicate experiments, and which contained at least one seed-match to one of the KSHV miRNAs. We identified 704 putative direct targets in DG-75 cells (Figure 3B), and 980 putative direct targets in EA.hy926 cells (Figure 3C). A complete list of putative direct targets can be found in Dataset S2 for DG-75 cells and in Dataset S3 for EA.hy926 cells. The overlap between the two datasets contained 153 putative direct targets (Dataset S4).

### Validation of putative KSHV miRNA targets

**Figure 4:**
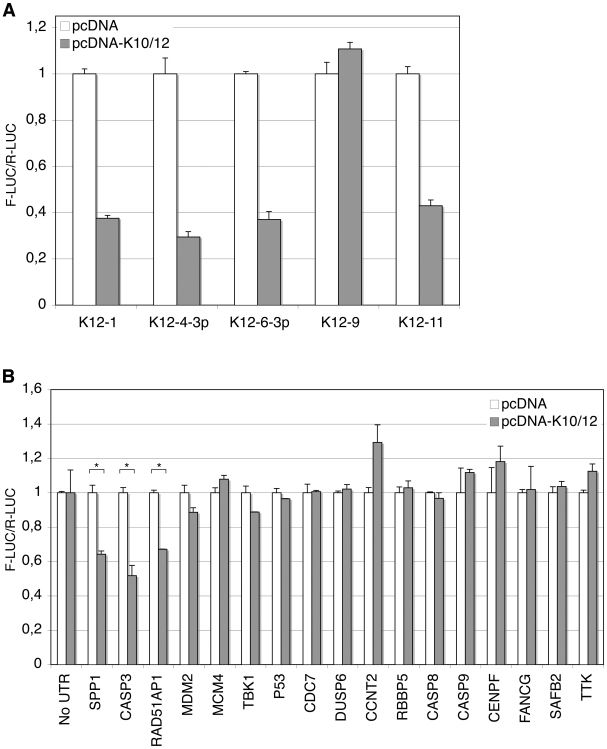
Validation of putative targets of KSHV miRNAs by luciferase assays. Luciferase assays were performed in triplicate 48 h post-transfection. The experiments were repeated at least 3 times, and one representative experiment is shown. **A.** KSHV miRNAs expressed from the pcDNA-K10/12 plasmid, but not miR-K12-9, can regulate the expression of sensor constructs containing complementary sequence to individual KSHV miRNAs. All differences but for miR-K12-9 were statistically significant (p<0.01). **B.** Dual-luciferase assay with psiCHECK-2 constructs containing either no UTR, or the indicated 3′ UTR. 293A cells were co-transfected with the luciferase construct and an empty pcDNA, or pcDNA-K10/12 construct. (* p<0.01).

In order to validate direct cellular targets of KSHV miRNAs, we turned to classical reporter assays in HEK293 cells (293A cells). We chose, among genes involved in pathways such as cell cycle regulation, DNA damage repair, and apoptosis, a subset of the 3′ UTR sequences identified as putative direct targets by our previous analysis. These candidates were then cloned 3′ to the firefly luciferase gene in the dual-reporter vector psiCHECK-2, also encoding a *Renilla* luciferase as a standard. We cloned and tested the full length 3′ UTR of sixteen candidate targets, which were tested in multiple independent assays. We first assessed that the K10/12 construct could repress the activity of luciferase sensors containing bulged complementary sequence (with a bulge at positions 9 to 12) to some of the KSHV miRNAs. For all of the KSHV miRNAs tested, except miR-K12-9, we could show a strong repression in the presence of pcDNA-K10/12 (Figure 4A). The lack of miR-K12-9 activity could relate to its lower expression in the context of the K10/12 construct (Tables S1 and S2). As opposed to what would have been expected based on the DG-75-K10/12 small RNA sequencing data, miR-K12-11 appeared to be functional in the FLP-K10/12, and we confirmed that it accumulated in higher amounts in these cells compared to the DG-75-K10/12 cells (data not shown). As a positive control for the luciferase assays with the selected putative targets, we used SPP1, a previously validated target of KSHV miRNAs [39]. The validation assays showed that only a subset of the 3′ UTRs tested resulted in a measurable repression of luciferase activity (Figure 4B). Among all the tested candidates, we observed the most important and reproducible down-regulation for two genes, Rad51AP1, involved in DNA damage repair, and Casp3, one of the main effectors involved in apoptosis induction. The RAD51AP1 reporter showed a down-regulation of 30 to 40% across luciferase experiments, while the Casp3 reporter showed a down-regulation of 40 to 50% (Figure 4B). We thus hypothesized that the anti-apoptotic phenotype of KSHV miRNA-expressing cells could be in part caused by the regulation of Casp3, and decided to continue this study by focusing on this protein.

### Targeting of Casp3 by miR-K12-1, K12-3 and K12-4-3p

**Figure 5:**
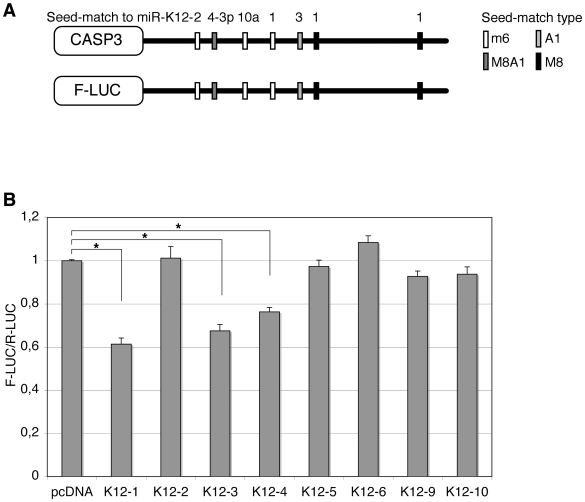
Caspase 3 is targeted by several KSHV miRNAs. Luciferase assays were performed multiple times in triplicate 48 h post-transfection. **A.** Schematic representation of Casp3 3′ UTR showing potential seed-matches for KSHV miRNAs. The seed-match types are described in the text. **B.** Dual luciferase assays performed in 293A cells with the Casp3 luciferase sensor and pcDNA constructs expressing the indicated individual miRNAs. Luciferase ratios relative to empty psiCHECK-2 set to 1 are displayed. (* p<0.01).

The initial analysis of Casp3 3′ UTR revealed 8mer or 7mer seed-matches [15] for miR-K12-4-3p (one M8A1 site), miR-K12-1 (two M8 sites), and miR-K12-3 (one A1 site). In addition, 6mer seed-matches to miR-K12-1, miR-K12-2 and miR-K12-10a could be found (Figure 5A). In order to further identify regions of the Casp3 3′ UTR that were susceptible to regulation by KSHV miRNAs, we subdivided the 3′ UTR in three parts and cloned them in the reporter vector. None of the tested fragments showed such a strong repression as the full-length sequence, suggesting that all putative miRNA binding sites are required for efficient repression, or that the binding sites function optimally only in their natural context (Figure S6). We then transfected pcDNA constructs expressing individual miRNAs (miR-K12-1 to -6, K12-9 and K12-10) to identify whether a single, or multiple miRNAs, mediated Casp3 regulation. We found that as suggested by the seed-matches quality, miR-K12-1, K12-3 and K12-4-3p (in decreasing order of repression observed) were able to significantly regulate the expression of the reporter fused to the 3′ UTR of Casp3 (Figure 5B). Expression of miR-K12-2 and K12-10, or of miRNAs with no predicted seed-matches (miR-K12-5, K12-6 and K12-9) had no effect on the Casp3 sensor.

**Figure 6:**
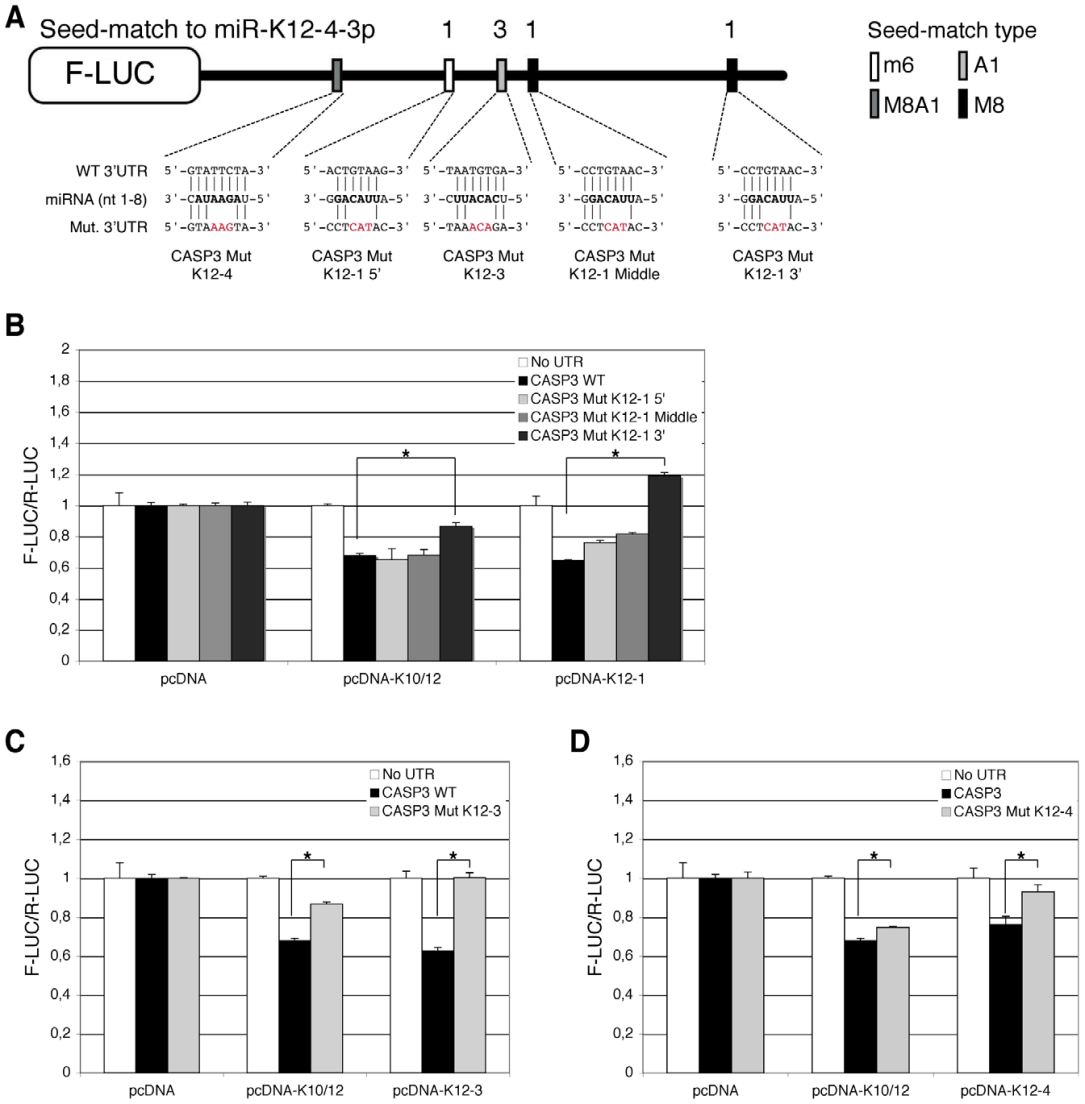
Identification of KSHV miRNAs binding sites in the 3′ UTR of Casp3 transcript by mutational analysis. Luciferase assays were performed multiple times in triplicate, 48 h post-transfection. **A.** Schematic representation of Casp3 luciferase sensor and of the mutagenesis performed within the potential binding sites of miR-K12-1, miR-K12-3 and miR-K12-4-3p. A mutant was generated for each potential miRNA binding site. Dual luciferase assays were performed with the Casp3 luciferase wild type (WT) or mutant sensors and pcDNA constructs expressing either the K10/12 construct or the individual miRNA miR-K12-1 (**B**), K12-3 (**C**) or K12-4-3p (**D**). Luciferase ratios relative to empty psiCHECK-2 set to 1 are displayed. (* p<0.01).

Subsequently, we aimed at determining which of the five putative binding sites for miR-K12-1, K12-3 and K12-4-3p were most important for Casp3 downregulation. To this end, we mutagenized each individual seed-match by introducing three point mutations to disrupt miRNA binding in the luciferase sensor containing Casp3 3′ UTR (Figure 6A). The resulting luciferase reporters were tested with miRNA expression constructs for either the 10 intronic miRNAs, or the individual miR-K12-1, K12-3 and K12-4-3p. As shown in Figure 6B, only the 3′ proximal binding site for miR-K12-1 appears to be functional, as the Casp3 Mut K12-1 3′ luciferase sensor could not be regulated by the pcDNA-K10/12 or the pcDNA-K12-1 constructs. The binding site for miR-K12-3 was also validated, as the mutant luciferase sensor for this miRNA is not regulated by the pcDNA-K10/12 or the pcDNA-K12-3 construct (Figure 6C). Finally, the binding site for miR-K12-4-3p was validated, although it seems to be less potent than the two others in terms of luciferase regulation (Figure 6D). In conclusion, we showed that Casp3 3′ UTR is regulated *via* three binding sites for (from 5′ to 3′) miR-K12-4-3p, K12-3 and K12-1. The positions of these sites explain why the luciferase assay done with the Casp3 3′ UTR fragments (Figure S6) did not reveal obvious differences as each individual fragment contained one of the three validated sites.

### KSHV miRNAs decrease endogenous Casp3 levels

**Figure 7:**
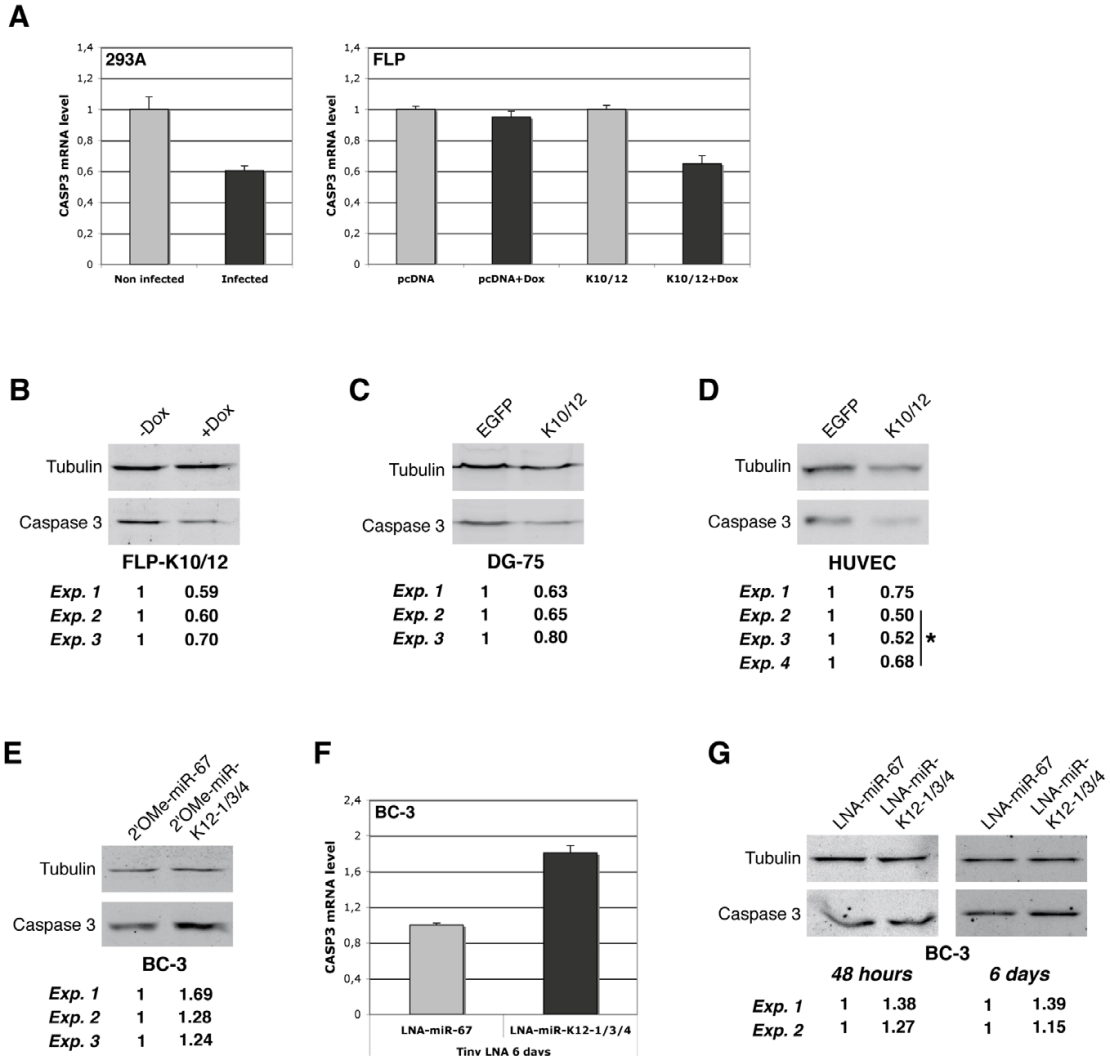
Endogenous Casp3 is regulated by KSHV miRNAs in different cell lines. **A.** qRT-PCR analysis of Casp3 mRNA expression in non-infected *vs. de novo* KSHV-infected HEK293 cells (left panel), and in inducible FLP-pcDNA or FLP-K10/12 cells (right panel), by comparing the non-treated *vs.* doxycycline-treated conditions. Error bars represent the standard deviation observed for 3 technical replicates. **B.** Western blot analysis and signal quantification from three independent experiments for Casp3 and Tubulin on the inducible FLP-K10/12 cell line, non-doxycycline-treated *vs.* doxycycline-treated conditions, and **C.** DG-75 cells expressing either EGFP or the K10/12 miRNA cluster. **D.** Western blot analysis and signal quantification from four independent experiments for Casp3 and Tubulin on primary or E6/E7 HUVEC cells stably expressing either EGFP or the K10/12 miRNA cluster after lentiviral transduction and antibiotic selection. *indicates experiments done in E6/E7 HUVEC cells. **E.** Western blot analysis and signal quantification from three independent experiments for Casp3 and Tubulin on KSHV miRNA inhibited-BC-3 cells. Cells were transfected with a 2′-O-methylated oligonucleotide antisense to the control cel-miR-67 (2′OMe-miR-67), or with a mix of oligonucleotides antisense to miR-K12-1, K12-3, and K12-4-3p (2′OMe-miR-K12-1/3/4) at the same final concentration, and harvested 48 h later. **F.** qRT-PCR analysis of Casp3 mRNA expression in KSHV miRNAs inhibited-BC-3 cells by tiny LNAs treatment. Cells were incubated for 6 days with 8mer LNA-oligonucleotides antisense to the seed region of the control miR-67 (LNA-miR-67), or with a cocktail of oligonucleotides antisense to miR-K12-1, -3, and 4-3p (LNA-miR-K12-1/3/4) at the same final concentration. **G.** Western blot analysis and signal quantification from two independent experiments for Casp3 and Tubulin on the same cells treated with the indicated tiny LNAs for 48 h (left panel) or 6 days (right panel).

In order to measure the effect of KSHV miRNAs on endogenous Casp3, we first performed real-time quantitative PCR analysis of 293A cells following primary infection and antibiotic selection of rKSHV infected cells [40]. We found that the level of Casp3 transcript decreased two fold following infection (Figure 7A, left panel). We also measured the level of Casp3 mRNA in the doxycycline-inducible FLP cells, and observed a similar down-regulation upon induction in FLP-K10/12 cells, but not in control FLP-pcDNA cells (Figure 7A, right panel). We then measured Casp3 protein levels in FLP-K10/12 and DG-75-K10/12 cells, and observed a significant down-regulation in three independent experiments (average of 0.63-fold, p = 0.0005 and 0.69-fold, p = 0.0046 respectively) (Figure 7B and C). We then turned to HUVEC endothelial cells, one of the two main cellular types infected *in vivo* by KSHV, and performed western blot analysis of primary or E6/E7 HUVEC cells stably transduced with either the EGFP, or the K10/12 lentiviral construct. In four independent experiments, the level of Casp3 protein was significantly down-regulated in K10/12 cells compared to the control EGFP cells (average of 0.61-fold, p = 0.0007) (Figure 7D).

In order to assess whether the down-regulation of Casp3 in naturally KSHV infected cells was caused by the specific presence of the three previously identified miRNAs, we used an antisense approach to inhibit specifically miR-K12-1, K12-3 and K12-4-3p. We thus employed either classical full-length 2′-O-methylated (2′OMe) antisense oligoribonucleotides [41], or short Locked Nucleic Acid oligonucleotides directed only against the seed of each individual miRNAs (tiny LNAs) [42]. In three independent experiments, transfection of a cocktail of 2′OMe oligonucleotides against miR-K12-1, K12-3 and K12-4-3p (2′OMe-miR-K12-1/3/4) in BC-3 cells resulted in a modest but measurable increase of Casp3 protein level compared to a control 2′OMe oligonucleotide (2′OMe-miR-67) (1.4-fold on average, p = 0.0486) (Figure 7E). The advantage of using tiny LNAs to inhibit miRNA function over the 2′OMe oligonucleotides is based on the fact that they do not require transfection to enter the cells. We therefore tested the inhibition efficiency of tiny LNAs on luciferase sensors in HEK293 cells and found that they could readily revert the targeted miRNA regulation (Figure S7). BC-3 cells grown in a medium containing a cocktail of tiny LNAs each directed against one of the three KSHV miRNAs listed above (LNA-miR-K12-1/3/4) also showed an 1.8-fold increase in Casp3 mRNA (Figure 7F) accompanied with a somewhat milder increase in the protein levels compared to control tiny LNA (LNA-miR-67) (1.3-fold on average, p = 0.0018) (Figure 7G; left panel for 48 h, and right panel for 6 days). Taking these results altogether, we can definitely conclude that Casp3 is regulated at both mRNA and protein levels by the KSHV-encoded miR-K12-1, K12-3, and K12-4-3p.

### Inhibition of KSHV miRNAs reduce apoptosis in infected cells

**Figure 8:**
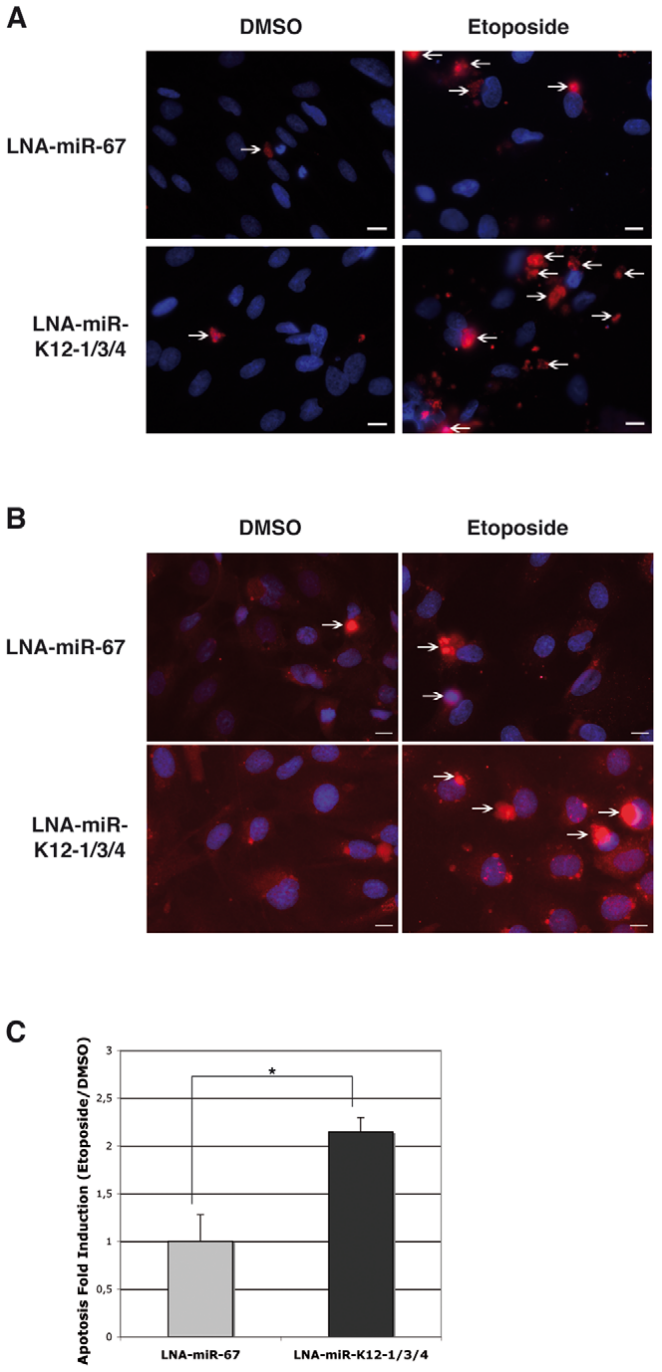
KSHV miRNAs inhibit apoptosis in KSHV-infected endothelial cells. **A**. Microscopic analysis following DAPI (blue) and cleaved Casp3 (red) staining of K-iLEC cells treated with DMSO or etoposide, and after KSHV miRNA inhibition by tiny LNA (LNA-miR-K12-1/3/4) or the control (LNA-miR-67). Arrows indicate cells that were counted as cleaved Casp3 positive. Bar size is 10 µM. **B.** Microscopic analysis following DAPI (blue) and TUNEL (red) staining of K-iLEC cells treated with DMSO or etoposide, and after KSHV miRNA inhibition by tiny LNA (LNA-miR-K12-1/3/4) or the control (LNA-miR-67). Arrows indicate cells that were counted as TUNEL positive. Bar size is 10 µM. **C.** Mean apoptosis fold induction measured from three independent TUNEL experiments; a significant difference of apoptosis induction (p = 0.0056) is observed between the LNA-miR-67 and the LNA-miR-K12-1/3/4 treatment.

In order to test the biological relevance of the repression of Casp3 by these KSHV-encoded miRNAs, we decided to look at Casp3 cleavage or its direct and indirect endogenous cleavage substrates, such as respectively Poly[ADP-ribose] polymerase-1 (PARP-1) or genomic DNA. We thus treated BC-3 cells with a cocktail of tiny LNAs directed against the three Casp3-targeting viral miRNAs, and measured PARP-1 cleavage following staurosporine treatment for 8 h. In the absence of staurosporine, inhibition of KSHV miRNAs had no or little effect on PARP-1 levels (Figure S8, left panel). Upon treatment, we found that cells pre-treated with anti-KSHV specific tiny LNAs (LNA-miR-K12-1/3/4), but not with the control tiny LNA (LNA-miR-67), accumulated slightly more of the PARP-1 cleavage product (Figure S8, right panel). We also tested the effect of this inhibition using KSHV-infected immortalized lymphatic endothelial cells (iLECs) by measuring the appearance of cleaved Casp3 and the extent of apoptosis-induced genomic DNA nicks following a 24 h etoposide treatment. iLECs represent one of the most relevant cell types implicated in KSHV pathogenesis [43]. We observed an increase in the number of cleaved Casp3 positive cells (Figure 8A) and TdT-mediated dUTP nick end labeling (TUNEL) positive cells (Figure 8B) over mock-treated (DMSO) controls when miR-K12-1, K12-3 and K12-4-3p were inhibited with the tiny LNA cocktail (LNA-miR-K12-1/3/4), over the control. In three independent experiments, the mean fold induction of etoposide-induced TUNEL positive cells (over the DMSO treated control) following inhibition of miRNAs (LNA-miR-K12-1/3/4) was significantly greater (2.30-fold, p = 0.041) than in cells treated with the control tiny LNA (Figure 8C). These data suggests that the KSHV-encoded miR-K12-1, K12-3 and K12-4-3p contribute to protection of etoposide-induced apoptosis in KSHV infected iLECs.

## Discussion

Viral miRNAs have only recently attracted attention in studies into viral genetics, and their importance during the course of infection remains to be fully established. Almost all of these miRNAs were found in viruses belonging to the herpesvirus family; viruses that are associated with latency and that suggest long-term disease progression. Like other members of the gammaherpesvirus subfamily, KSHV is associated with a number of neoplastic disorders including Kaposi's sarcoma and B-cell lymphomas [3]. Some cellular targets of KSHV miRNAs have been previously reported. For example, miR-K12-11 has been shown to target a subset of genes that are also targeted by its homologous human miRNA, miR-155, that shares an identical seed region with this miRNA [26], [27]. Among the validated targets of miR-K12-11 are two transcription factors, BACH1 and Fos. Although Fos itself provides a potential link between KSHV infection and oncogenesis, the authors did not show that KSHV miRNAs directly participate in cancer progression. The study of Samols and colleagues identified another potential candidate as a KSHV miRNA target that could contribute to cell transformation [39]. Indeed, with a microarray-based approach similar to the one that was used in this study, they found that thrombospondin (THBS1), a gene involved in angiogenesis, is regulated by KSHV miRNA expression. However, the analysis was performed in HEK293 cells, which are not representing cells naturally infected by KSHV. More recently, the Ganem laboratory also reported on the identification of cellular targets of KSHV miRNAs using a transcriptomic-based approach, with the Bcl2-associated factor BCLAF1 as one of the identified targets of several KSHV miRNAs [44]. Other targets of KSHV miRNAs that have been identified very recently are p21, IκBα, TWEAKR and Gemin 8 [35], [45]–[47].

The aim of this study was to define the role played by KSHV miRNAs in apoptosis inhibition. The apoptotic processes can be executed intracellularly by the release of various factors (e.g. cytochrome c or SMAC/DIABLO) from mitochondria, or extracellularly through transmembrane death receptors, which are activated by their ligands. In both the intrinsic and extrinsic pathways, caspases are recruited and activated, and in turn they cleave substrates leading to the execution of apoptosis. In the intrinsic pathway, cytochrome c leaks from mitochondria [48], and binds to the adaptor apoptotic protease activating factor-1 (APAF1) to form the multi-protein structure, coined the apoptosome. The latter recruits Casp9, which in turn activates downstream effector caspases 3, 6 and 7 [49]. In the extrinsic pathway, ligands such as TRAIL and FasL activate specific pro-apoptotic death receptors at the cell surface [50]–[52], which results in the binding of the intracellular domains of the receptors to the adaptor protein Fas-associated death domain [53]. This leads to the assembly of the death-inducing signaling complex DISC, and to the recruitment of initiator caspases 8 and 10 [54]. Upon stimulation of these two caspases, effector caspases 3, 6 and 7 are activated. Thus, the intrinsic and extrinsic pathways converge at the level of the effector caspases, which highlights Casp3 as a critical factor in the control of apoptosis. In this study, we observed that KSHV miRNAs have a negative effect on apoptosis, as HEK293 cells and DG-75 B lymphocytes expressing these viral miRNAs are partially protected from apoptosis induction by staurosporine. We also measured Casp3 activity in the HEK293 cells, and showed that the presence of KSHV miRNAs resulted in a sharp decrease of Casp3/7 activity upon staurosporine induction. While our data does not rule out that the observed effect in HEK293 cells is due to a decreased activity of Casp7, the evidence available to date indicates that Casp3 activity is predominant over Casp7 activity, and that Casp3 is likely the major executor of apoptosis [55]. However, we were unable to monitor the effect of KSHV miRNA on Casp3/7 activity in DG-75 cells. Indeed, these cells are resistant to caspase activation by the intrinsic pathway [36], and accordingly, we could not induce Casp3/7 cleavage with staurosporine. This result confirms that Annexin V levels do not only measure caspase-dependent apoptosis, and therefore indicates that KSHV miRNAs are regulating both caspase-dependent and -independent apoptosis.

To discover cellular targets of KSHV miRNAs, we used a microarray-based approach to identify transcripts regulated by KSHV miRNAs in both the B lymphocyte DG-75 cell line and the endothelial EA.hy926 cell line. Based on their expression profiles, the samples primarily clustered according to the cell line (DG-75 or EA.hy926), and, within these two clusters, according to the presence of KSHV miRNAs. Using small RNA deep-sequencing data, we determined the relative abundance of each miRNA within the expressed cluster, which enabled us to show that transcripts containing seed-matches to KSHV miRNAs within their 3′UTR were significantly more down-regulated that transcripts without such binding sites. This enabled us to generate a list of putative targets to follow in further functional assays. Our validation rate was relatively low, reflecting presumably the fact that many miRNAs (virus-encoded and endogenous) changed in these experiments, leading to complex secondary effects. We looked for seed-match sites within the coding sequences of down-regulated transcripts and could identify a few (listed in Dataset S1), but the validation of these sites can prove challenging. Nevertheless, we validated two candidate targets that are biologically relevant for KSHV infection, Rad51AP1 and Casp3. Rad51AP1 is a DNA binding protein that participates in RAD51-mediated homologous recombination, and is important for the preservation of genome integrity [56]. Because KSHV has been shown to induce DNA damage response through the expression of v-cyclin [57], the down-regulation of Rad51AP1, which will require further validation, might be important in the context of viral infection. In light of our initial aim to define the role of KSHV miRNAs in apoptosis inhibition, we focused our efforts on the characterization of Casp3 as a target of KSHV miRNAs. We confirmed that a Casp3 3′ UTR luciferase reporter construct is regulated by three KSHV miRNAs, and we identified three miRNAs, miR-K12-1, miR-K12-3 and miR-K12-4-3p, as being responsible for this regulation, as well as their binding sites within Casp3 3′ UTR.

We then showed that endogenous Casp3 was also regulated by KSHV miRNAs, both at the mRNA and protein levels, and in different cell types. We also showed that inhibition of miR-K12-1, K12-3 and K12-4-3p in KSHV-infected cells resulted in an upregulation of Casp3 expression, which in turn translated into an increase in apoptosis, as assessed by cleaved Casp3 quantification and TUNEL assay analysis. These findings are consistent with a report that described the role of KSHV in conferring a survival advantage to endothelial cells [58]. In this report, Wang et al. showed that the level of Casp3 activity was decreased in KSHV-infected HUVEC cells subjected to staurosporine treatment (or other apoptotic insults). The regulation of Casp3 is not the only explanation for KSHV miRNAs-mediated inhibition of apoptosis, especially since we showed that caspase-independent apoptosis was also affected. It is of course highly probable that other factors in the apoptosis pathway are also targeted by KSHV miRNAs. For example, Abend *et al.* recently reported that KSHV miR-K12-10 targeted the TNF-like weak inducer of apoptosis (TWEAK) receptor [47], which indicates another level of regulation of one certain type of apoptosis.

In summary, our findings demonstrate that KSHV miR-K12-1, K12-3 and K12-4-3p target the effector caspase 3. The down-regulation of Casp3 by KSHV miRNAs results in a decrease in apoptosis activity in different cell types including endothelial cells that are biologically relevant for KSHV infection *in vivo*. The specific inhibition of these miRNAs in infected cells increased Casp3 levels and cell death. Apoptosis is frequently inhibited in tumor cells, and our results are in agreement with a recent report that indicates that the active form of Casp3 is detected less frequently in Kaposi sarcoma lesions in patients from Brazil [59]. Our data therefore suggests that apoptosis regulation by the viral miRNAs could contribute to the malignant phenotype triggered by KSHV infection. In the long term, delivery of specific inhibitors of these viral miRNAs in KSHV-infected patients to restore apoptotic clearance of the virus by the immune system could be an interesting novel therapeutic approach.

## Material and Methods

### Cell lines

DG-75 and BCBL-1 cells (obtained through the NIH AIDS Research and Reference Reagent Program (Cat# 3233 from McGrath and Ganem)) were grown in RPMI 1640 medium containing 10% fetal calf serum (FCS), 100 UI/mL penicillin, 100 µg/mL streptomycin and 2 mM L-Glutamine. BC-3 cells (ATCC) were grown in the same media with 50 µM ß-Mercaptoethanol. EA.hy926, QBI-HEK 293A (QBiogene), Flp-In T-REx-293 (Invitrogen), and HEK293 cell lines were grown in DMEM supplemented with 10% FCS and penicillin/streptomycin. Primary and E6/E7 HUVEC cells (from Promocell) were cultured in a humidified 5% CO_2_ atmosphere at 37°C in endothelial basal medium (Promocell) supplemented with 10% FCS, gentamicin, amphotericin and supplement kit provided with the media. To obtain immortal lymphatic endothelial cells (iLECs) primary human LEC cells (Promocell) were immortalized by the HPV oncogenes E6/E7 as previously described (Moses et al., 1999). iLEC cells were maintained in endothelial basal medium (Promocell) supplemented with 5% human AB serum (HS; Sigma, St. Louis, Mo.).

### KSHV infection of iLECs

Wildtype KSHV was produced from BCBL-1 cells induced with 20 ng/mL PMA. The virus-containing supernatant was collected after three days by ultracentrifugation (21,000 rpm at 4°C for 2 h), and resuspended in TNE buffer (150 mM NaCl, 10 mM Tris pH 8, 2 mM EDTA, pH 8). For the KSHV infection iLEC cells were plated in 6-well plates one day before the infection using multiplicity of infection (MOI) 1 in the presence of 8 µg/mL polybrene (Sigma). The infection was performed as spin-infection by centrifugation at 2500 rpm (Heraeus Multifuge 3 S-R; Thermo Scientific) for 30 min at room temperature. Cells were then returned to 37°C, 5% CO_2_, and after 4 h of incubation fresh complete media was added. The virus-containing medium was removed the next day, and replaced with fresh complete media. The extent of KSHV infection was monitored by expression of the latent nuclear antigen-1 LANA-1 in the nuclei of KSHV-infected cells (K-iLECs) and detected by immunofluorescence using anti-LANA antibody (13-210-100, Advanced Biotechnologies Inc).

### Primary rKSHV infection of HEK293 cells

rKSHV.219 infected HEK293 cells were reactivated by incubating them in DMEM medium containing 1 mM sodium butyrate and 20 ng/mL TPA (tetradecanoyl phorbol acetate) for 24 h, and four more days with media containing sodium butyrate only. The supernatant was collected, filtrated through 0.45 µM filter, and 8 µg/mL polybrene was added before adding the supernatant to QBI-HEK 293A cells seeded one day before. After 4 h, the medium was replaced and the cells grown at 37°C for 2 days. As soon as green fluorescent started to appear, 1 µg/mL puromycin was added to the medium. Cells were harvested for RNA analysis after at least 21 days under puromycin selection.

### Generation of stable cell lines expressing KSHV miRNAs

Cell lines stably expressing the ten intronic KSHV miRNAs were generated using the “Virapower” lentiviral transduction system with the vector pLENTI6/V5 (Invitrogen) and Gateway cloning. The miRNA encoding intronic region was amplified by a two-step PCR using cDNA prepared from KSHV infected BCBL-1 cells (PCR primers: KSHV miRK_for and KSHV miRK_rev for the first PCR and attB1_external for and attB2_external rev for the second PCR), cloned into pDONR207 and transferred to pLENTI6/V5-DEST (Invitrogen). PCR primers are provided in Table S3. The control lentiviral vector pLENTI6/V5-EGFP was a kind gift from Oliver Rossmann. In order to generate lentiviruses for transduction of cells with KSHV miRNAs, the ViraPower Lentiviral Gateway Expression System (Invitrogen) was employed according to the manufacturer's instructions. The packaging mix contained plasmids pLP1, pLP2 and pLP/VSVG. Virus-containing medium was cleared with a 0.45 µm filter and added with polybrene (8 µg/mL) to DG-75 (1×10^6^ cells/mL) or EA.hy926 (3×10^5^ cells/mL) target cells for transduction. In the case of EA.hy926 cells, the plates were centrifugated 30 min at 2500 rpm to increase transduction efficiency. Two days after transduction, when the EGFP signal in the control cells became visible, Blasticidin (1 µg/mL) was added to the medium to select for the transgene and gradually raised to a final concentration of 7.5 µg/mL for DG-75 cells and 3 µg/mL for EA.hy926 cells after six days. Efficiency of selection was determined by analyzing the proportion of EGFP expressing control cells by fluorescence activated cell sorting (FACS). Cell lines were used for experiments when 100% of control cells expressed EGFP.

Primary HUVEC cells were transduced with lentiviruses (pLenti6-vector; Invitrogen) encoding EGFP or 10/12 KSHV miRNA cluster (K10/12) and maintained under blasticidin selection (5 µg/mL) in endothelial basal medium supplemented as above. The cells were replenished with fresh medium every second day and passaged when necessary.

### Generation of inducible FLP-293 stable cell lines

The Flip-In stable cell lines were generated using the Flp-In T-REx-293 cell line (Invitrogen) and according to the manufacturer's instructions (Invitrogen). Briefly, cells were seeded one day before at 10^6^ cells/well in 6-well plates. Cells were transfected with 3.6 µg and 0.4 µg respectively of pOG44 (Invitrogen) and each pcDNA for each cell line with lipofectamine 2000 (Invitrogen). The media was replaced 24 h after transfection, and cells were passaged into 10 cm dishes 24 h later to achieve a desired confluency of maximum 25% prior selection. Hygromycin (Invivogen) was added at a concentration of 200 µg/mL and then raised 2–3 days later at a concentration of 250 µg/mL. The media was replaced each 3–4 days until 2–3 mm wide foci appeared. Cells were then passaged into 75 cm^2^ flasks for amplification. Efficiency of the selection was then assayed by β-galactosidase staining, for the loss of β-galactosidase activity, and/or by northern blot for the detection of the miRNA.

### Computational microarray analysis

The microarray data were submitted to the gene expression omnibus database (http://www.ncbi.nlm.nih.gov/geo) under the accession number GSE18946. We imported the CEL files into the R software (R Development Core Team (2008). R: A language and environment for statistical computing. R Foundation for Statistical Computing, Vienna, Austria. ISBN 3-900051-07-0, http://www.R-project.org) using the BioConductor affy package [57]. The probe intensities were corrected for optical noise, adjusted for non-specific binding and quantile normalized with the gcRMA algorithm [58].

Per gene log2 fold change was obtained through the following procedure. We first fitted a lowess model of the probe log2 fold change using the probe AU content. We used this model to correct for the technical bias of AU content on probe-level log2 fold change reported by [59]. Subsequently, probe set-level log2 fold changes were defined as the median probe-level log2 fold change. Probe sets with more than half of the probes (6) mapping ambiguously (more than 1 locus) to the genome were discarded, as were probe-sets that mapped to multiple genes. We then collected all remaining probe sets matching a given gene, and averaged their log2 fold changes to obtain an expression change per gene. For sequence analyses, we selected for each gene the RefSeq transcript with median 3′ UTR length corresponding to that gene.

Controls and transductions were performed in triplicates in both cell lines (DG-75, EA.hy926), and we used limma [60] to compute differential regulation p-values. Finally, for each cell line, we only analyzed genes which had at least one probe set that was called present in either all replicates of miRNA transduction or all replicates of the control (or both).

#### Transcriptome-wide regulatory effect of the KSHV miRNAs

To determine whether transduction of the KSHV miRNAs had the expected effect on mRNA expression, we first computed a KSHV miRNA sensitivity score for each mRNA. This was defined as the sum over all KSHV miRNAs, the number of seed matches to the KSHV miRNA in the 3′ UTR weighted by the relative abundance of the KSHV miRNA. The KSHV miRNA expression was determined in DG-75 cells by Illumina small RNA sequencing and the relative abundance of a miRNA was defined as the number of reads that mapped to this KSHV miRNA divided by the total number of reads that mapped to any KSHV miRNA. Because the relative abundance of KSHV miRNAs was comparable in DG-75 and EA.hy926 cells (Table S1 and Figure S4), we used the same KSHV miRNA sensitivity score to analyze the DG-75 and the EA.hy926 microarrays. The Human Genome U133 Plus 2.0 Affymetrix microarrays used in this study measure the expression of a total of 15678 genes. Of those, we selected the 1000 genes with the highest KSHV miRNA sensitivity score as the most likely targets of KSHV miRNAs. Of those, 611 genes were actually expressed in DG-75 cells and 674 in EA.hy926 cells. We compared the fold change of these genes with those that had no KSHV miRNA seed match in their 3′ UTR (2047 expressed genes in DG-75 and 2137 expressed genes in EA.hy926) using a two-sided Wilcoxon rank sum test. The bar plots in Figure 3A represent the mean and two standard errors around the mean fold change of KSHV sensitive mRNAs and mRNAs with no seed matches in their 3′ UTR.

To test whether the 3′ UTR length could alone explain the downregulation of the KSHV sensitive mRNAs in cells transduced with KSHV miRNAs, we sampled 1000 genes in such way that their 3′ UTR length distribution was identical to that of the KSHV sensitive mRNAs. For each cell line, we then computed the average fold change of the subset of these 1000 genes that were actually expressed. Finally, we repeated this procedure 1000 times. The mean fold change and two standard errors around the mean over these 1000 randomizations are reported on Figure 3A (blue bars).

#### List of putative direct targets

Transcripts that are direct targets of the intronic KSHV miRNAs would ideally carry at least one seed match to at least one of the intronic KSHV miRNA, and it should be down-regulated in the KSHV miRNAs transduction compared to the EGFP control transduction.

We considered that an mRNA was down-regulated if its log2 fold change was negative and the limma p-value of differential regulation smaller than 0.05. In addition, we only considered genes for which the magnitude of down-regulation was between 40% and 300% (log2 fold M: −2<M<−0.5). Through applying these criteria, we generated three lists of putative direct targets: one for the DG-75 cells, one for the EA.hy926 cells, and then intersected these two lists to generate a list of putative direct targets that are common to DG-75 and EA.hy926.

### Generation of inducible miRNA expression and luciferase sensor vectors

To generate the pcDNA-K10/12, the KSHV intronic miRNA cluster was PCR-amplified from BAC36 DNA [60] and ligated into the *Bam* HI and *Xho* I sites of the pcDNA5/FRT/TO (Invitrogen). The primer sequences were (sense and antisense primers are indicated in respective order): 5′-ATATGGATCCGAATGCGTGCTTCTGTTTGA, 5′-ATATCTCGAGTTTACCGAAACCACCCAGAG. The empty pcDNA vector was obtained by digesting the pcDNA-K10/12 with *Pme* I, followed by ligation of the plasmid. For KSHV miRNA individual expression vectors, a region of approximately 300 nt surrounding each pre-miRNA (or the miRNA cluster) was PCR-amplified from BAC36 DNA. *att*B1/2 sequences were added by nested PCR and the resulting PCR product were cloned into pDONR207 (Invitrogen) and then recombined in pLenti6/V5-DEST using Gateway technology (Invitrogen). The *att*B1/2 primer sequences are (sense and antisense primers are indicated in respective order): 5′-ACAAGTTTGTACAAAAAAGCAGGCT, 5′-ACCACTTTGTACAAGAAAGCTGGGT. The specific primers are indicated in Table S3. The individual miRNA expression cassettes were then subcloned *via* PCR amplification from pLenti6/V5-DEST expressing vectors and ligated into the *Xho* I and *Apa* I sites of pcDNA5/FRT/TO, and with the primers indicated in Table S3. To generate luciferase reporter plasmids, psiCHECK-2 (Promega) was modified by inserting the Gateway cassette C.1 (Invitrogen) at the 3′-end of the firefly luciferase gene into the *Xba* I site of psiCHECK-2. *att*B-PCR products were cloned into pDONR/Zeo (Invitrogen) and recombined in the modified psiCHECK-2 vector by Gateway cloning. The 3′ UTR sequence of the different candidates were obtained from the Ensembl database (www.ensembl.org) and were nested PCR-amplified from QBI-HEK 293A cells' genomic DNA with the primers indicated in Table S3 and attB1/2 primers. The imperfect match sensors for KSHV miRNA were obtained by annealing the oligonucleotides indicated in Table S3 and PCR-based addition of the *att*B sequences using *att*B1/2 primers. The resulting PCR product was then cloned by Gateway recombination sequentially in pDONR/Zeo and psiCHECK-2 plasmids.

### Mutagenesis of Casp3 luciferase sensor

Mutagenesis was performed using QuikChange Lightning Site-Directed Mutagenesis Kit (Agilent Technologies) according to the manufacturer's instructions and using the oligonucleotides indicated in Table S3. Briefly, we mutagenised in the Casp3 luciferase reporter construct the nucleotides predicted to pair to position 3 to 5 of the miRNA sequence to prevent pairing of the miRNA seed sequence on Casp3′s predicted target sites.

### Luciferase assays

QBI-HEK 293A cells were seeded in 48-well plates at 10^5^ cells/well and then incubated a few hours. When cells were adherent, co-transfection of 25 ng of the reporter constructs and 250 ng of the pcDNA-K10/12 (or pcDNA as control vector) were performed using Lipofectamine 2000 (Invitrogen). After 48 h, cells were then washed in PBS and lysed with 65 µL of passive lysis buffer (Promega), and 10 µL were assayed for firefly and *Renilla* luciferase activity, using the dual-luciferase reporter assay system (Promega) and a luminescence module (Glomax, Promega). The relative reporter activity was obtained by first normalizing to the transfection efficiency with the *Renilla* activity, and then, to the firefly activity obtained for the empty control reporter, in presence of the pcDNA-K10/12 or pcDNA, to normalize for the effect of transfection of these expression vectors.

### Western blot analysis

For Western blot analysis of HUVEC cells, cells were extracted in ELB lysis buffer (150 mM NaCl; 50 mM HEPES, pH 7.4; 5 mM EDTA and 0.1% NP40) and 30 µg of proteins was separated on 12% SDS-PAGE and transferred on to nitrocellulose membranes according to standard protocols. Primary antibodies used in Western blotting were anti-caspase-3 (MAB4603; Millipore) and anti-γ-tubulin (GTU-88; Sigma-Aldrich). HRP-conjugated anti-mouse (AP308P; Chemicon) immunoglobulin was used as a secondary antibody. Filters were visualized on SuperRX film (Fuji) using the ECL chemiluminescence system (Pierce, Rockford, IL). The intensity of the chemiluminescence signals was quantified with FluoChem 880 imager and software (Alpha Innotech Corporation).

For Western blot analysis of DG-75, BC-3 or FLP-293 cells, cells were extracted in passive lysis buffer (50 mM Tris, 150 mM, NaCl, 5 mM EDTA and 0.5% NP40, 10% Glycerol and 10 µM MG132) and 15 µg or 45 µg of proteins, respectively from BC-3 or FLP-293 cells, was separated on 10% SDS-PAGE for PARP analysis, or on 15% SDS-PAGE for Casp3 analysis, and transferred on to nitrocellulose membranes according to standard protocols. Primary antibodies used in Western blotting were anti-caspase-3 (06-735; UpState), anti-PARP-1 [61] and anti-γ-tubulin (GTU-88; Sigma-Aldrich). IRDye 800CW-conjugated anti-rabbit and anti-mouse (926-32213 and 926-32212; Li-Cor Biosciences) immunoglobulins were used as secondary antibodies. The intensity of the fluorescence signals was quantified with Odyssey Infrared Imaging system and Odyssey v3.0 software (Li-Cor Biosciences).

### Northern blot analysis

RNA was extracted using Trizol reagent (Invitrogen) and Northern blotting was performed on 5 to 10 µg of total RNA as described before [23], [62]. Probes were 5′ ^32^P-radiolabelled oligodeoxynucleotides antisense to the miRNA sequence or to part of the U6 snRNA sequence. Blots were analyzed and quantified by phosphorimaging using a FLA5100 scanner (Fuji).

### Small RNA cloning and sequencing

Small RNA cloning was conducted from 50 µg of DG-75-K10/12 total RNA as previously described [63]. Small RNA sequencing was performed at the Institut de Génétique et de Biologie Moléculaire et Cellulaire (IGBMC, Illkirch, France) using an Illumina Genome Analyzer II with a read length of 36 base pairs (bp).

### Processing and annotation of small RNA sequences

An in-house Perl analysis pipeline was used to analyze the data produced by small RNA sequencing. After 3′ adaptor removal and size selection (exclusion of trimmed reads shorter than 15 nt), non-redundant sequences were mapped to the genomes from which they may derive and to other RNAs already annotated, using Nexalign (http://genome.gsc.riken.jp/osc/english/software/src/nexalign-1.3.5.tgz) permitting up to 2 mismatches. The *Homo sapiens* and KSHV genome sequences were respectively downloaded from the UCSC repository (assembly version hg19) and the GenBank database. The following sources of annotated transcripts were used: miRBase v.16 for miRNAs, GenBank v.180 for *Homo sapiens* rRNA, tRNA, sn-snoRNA, scRNA and piRNA, and Repbase v.16.01 for *Homo sapiens* and common ancestral repeats. By doing so, small RNAs that mapped unambiguously to sequences from one single functional category were easily classified, while the other ones were identified by applying this annotation rule based on the abundance of various types of sequences in the cell: rRNA > tRNA > sn-snoRNA > miRNA > piRNA > repeat > pathogen genome > host genome > unknown.

### cDNA synthesis and quantitative real-time PCR

#### miRNAs

Semi-quantitative real-time PCR for KSHV miRNAs was performed using the Light Cycler System (Roche) with a modified protocol from Shi and Chiang [64]. Briefly, total RNA was extracted with Trizol (Invitrogen) and provided with a 3′ poly-A-tail (Poly-A-Tailing Kit, Ambion). After phenol-chloroform extraction and ethanol-acetate precipitation, first strand synthesis with the anchor primer Poly(t)adpt was performed using Superscript II reverse transcriptase (Invitrogen). Quantification of miRNAs was performed using the FastStart DNA MasterPlus SYBR Green I Master Mix (Roche) with specific primer and AdptRev-primer. The PCR program was composed of an initial activation step for the Taq polymerase at 95°C for 10 min followed by 45 cycles of 95°C for 10 sec, 68°C for 5 sec (ΔT = 20°C/sec each) and 72°C for 6 sec (ΔT = 5°C/sec). 5.8S RNA was quantified and the results used for normalization. The changed levels of miRNA transcripts (relative to 100%) were calculated based on the empiric formula “level(%) = 1,8∧ΔCt”, based on quantification of synthetic miRNAs (data not shown). Primers used are shown in Table S3.

#### Caspase 3

To monitor the expression of Casp3 mRNA levels, cDNA was prepared from total RNA using Superscript II (Invitrogen). Transcripts were quantified by TaqMan PCR using the ABI Prism 7000 sequence detection system (Applied Biosystems). TaqMan probes were taken from the Universal Probe Library (Roche) and selection of probe-primer combinations was performed using the Assay Design Centre (Roche, www.universalprobelibrary.com). The PCR program was composed of a denaturation step at 94 °C for 12 min followed by 45 cycles of 95°C for 20 sec and 60°C for 1 min 72°C for 6 sec (ΔT = 5°fC/sec). HPRT-transcript was used for normalization of Ct-values. The changed levels of Casp3 transcripts (relative to 100%) were calculated based on the empiric formula “level(%) = 2∧ΔCt”.

The primers used were: RT_CASP3_for, RT_CASP3_rev and Roche universal probe #68; RT_HPRT_for, RT_HPRT_rev and Roche universal Probe #73 (sequences can be found in Table S3).

### BC-3 cells miRNAs inhibition with 2′O-methylated or LNA oligonucleotides

For inhibition of miRNAs, BC-3 cells were cultured in 6-well dishes and transfected with the 2′O-methylated oligonucleotides (provided by G. Meister) against individual KSHV miRNAs using Oligofectamine (Invitrogen). Oligonucleotides were used at a final concentration of 60 nM and transfections were performed according to manufacturer's instructions. Total proteins were extracted for analysis 48 h after transfection.

For inhibition of miRNAs with tiny LNAs, 2×10^6^ BC-3 cells were seeded in 6-well plates and incubated with the inhibitors (Table S3) against individual KSHV miRNAs or the control *C. elegans* miR-67. Oligonucleotides were used at a final concentration of 1.5 µM and incubated in the medium for 48 h, or 6 days by replacing twice the medium (day 2 and 5), prior to harvesting the cells.

### Annexin V and Casp3/7 activity cell death assays

The effects of the KSHV miRNAs on apoptosis were analysed by both measurement of caspase 3/7 activity and Annexin V/propidium idiode (PI) staining. Cell death was induced by adding 2 to 5 µM staurosporine (Sigma) for 8 h; DMSO was used as a control. For Annexin V binding analysis, 10^5^ HEK293 cells were seeded in 12-well plates, incubated overnight prior to addition of staurosporine or DMSO. Cells were harvested by trypsinization, washed in PBS, and resuspended in binding buffer (10 mM Hepes/NaOH (pH 7.4), 140 mM NaCl, 2.5 mM CaCl_2_) containing Annexin V conjugated with Allophycocyanin diluted at 1/100 (BD Biosciences, Le Pont-de-Claix France) and 2 µg/mL PI (Sigma-Aldrich, Lyon, France). The cells were incubated for 15 min in the dark and analyzed with a FacsCalibur flow cytometer (Becton Dickinson, Le Pont-de-Claix, France).

Statistical analysis for Annexin V geo means collected in individual experiments were performed using a Wilcoxon signed-paired rank test, as distribution of measurements in each condition did not fit normality tests. Differences were considered significant when p<0.05.

For caspase 3/7 activity assay, 2.5×10^4^ cells were seeded in 96-well plates, and staurosporine or DMSO immediately added. Caspase 3/7 activity was then measured using Caspase-Glo 3/7 Assay Kit (Promega) and normalized to the protein concentration determined by DC Protein Assay (Bio-Rad).

### Cleaved Casp3 quantification and TUNEL assay

KSHV infected immortalized (by stable expression of HPV16 E6/E7) human Lymphatic Endothelial Cells (K-iLEC) were seeded one day before at 5×10^4^ cells/well on 24-well plates. For inhibition of miRNAs, K-iLEC cells were treated with two doses of Tiny LNA oligonucleotides (48 h+48 h) at a final concentration of 1,5 µM. Apoptosis was induced with 500 µM Etoposide (Sigma Aldrich) and DMSO was used as a vehicle control (mock). Cells were fixed with 4% Paraformaldehyde (EMS, Hatfield, PA) 24 h after the treatment with Etoposide or mock. Coverslips were blocked 30 minutes with 5% goat serum and incubated first with 1∶800 diluted Cleaved Caspase-3 (Asp175) rabbit monoclonal antibody (Cell signaling) for 1 h at room temperature, then with a 1∶1000 dilution of a goat anti-rabbit secondary antibody coupled to Alexa Fluor 594 (Invitrogen). Alternatively, apoptosis was detected with TdT-mediated dUTP nick end labeling (TUNEL) assay according to manufacturer's instructions of the kit (In situ Cell Death Detection Kit, TMR red, Roche, Mannheim, Germany). The fluorochromes were visualized with a Zeiss Axioplan 2 fluorescent microscope (Carl Zeiss, Oberkochen, Germany). Images were acquired with a Zeiss Axiocam HRc, using Zeiss AxioVision (version 4.5 SP1) and Adobe Photoshop software (version 7.0; Adobe, San Jose, CA).

## Supporting Information

Dataset S1 Microarray analysis summary. Each row in the table represents one of the 15768 genes monitored by the Affymetrix arrays. The “RefSeq mRNA”, “Gene Name”, “Entrez Gene ID”, “gene description” and “mRNA annotation” columns contain the same information as in Dataset S2, S3 and S4 (see below). The “KSHV sensitivity 3′ UTR” and “KSHV sensitivity CDS” columns contain the *KSHV miRNA sensitivity score* described in the methods for the 3′ UTR and the Coding Region, respectively. The “mRNA presence DG-75” and “mRNA presence EA.hy926” columns indicate whether the gene was called present by the Affymetrix arrays in each cell line. The “3′ UTR hits” and “CDS hits” then indicate how many matches to the KSHV miRNAs were found in the 3′ UTR and in the Coding Region. The remaining fields (log2 mRNA fold change, log2 intensity, differential expr. P-value) are defined as in Dataset S2, S3 and S4 (see below).
(XLS)

Dataset S2 Putative direct targets of the KSHV miRNAs in DG75 cells. Each row in the table corresponds to one gene, identified by a representative RefSeq mRNA ID, a set of Affymetrix probes designed to monitor the expression of that gene, the Entrez Gene ID, the gene name and the gene description provided by NCBI RefSeq. The “mRNA annotation” field provides information about the length and the span of the Coding Domain of the representative mRNA used for the analysis. “log2 fold change K10/12 vs EGFP” contains the log2 fold changes in gene expression upon transducing the K12/10 construct compared to the EGFP control, while “log2 intensity” is the average signal intensity on the microarrays. “diff. Expression p-value” is the (uncorrected) P-value of differential expression as computed by the limma algorithm. The “Detectable” field is true whenever the gene could be detected in at least one of the samples from the corresponding cell line. Finally, the two “Seed matches to K10/12 miRNAs” field indicates the KSHV miRNAs for which at least one match to the seed recognition motif could be found in the 3′ UTR of the representative RefSeq mRNA. The procedures we used to build this table are described in the “Methods” section.
(XLS)

Dataset S3 Putative direct targets of the KSHV miRNAs in EA.hy926 cells. For details, please refer to Dataset S2 legend.
(XLS)

Dataset S4 Putative direct targets of the KSHV miRNAs in both cell lines. For details, please refer to Dataset S2 legend.
(XLS)

Figure S1 Northern blot analysis of BCBL-1, BC-3 and FLP-pcDNA and FLP-K10/12 cells grown in doxycycline-containing medium (final concentration of 1 µg/ml). U6 was used as a loading control.
(TIF)

Figure S2 The relative abundance of KSHV miRNAs is similar in K10/12 transduced DG-75 cells and K10/12 EA.hy926 cells. Each dot on the scatter represents one KSHV miRNA whose expression in KSHV-infected BCBL1 cells, DG-75 cells and EA.hy926 was quantified by qPCR. Plotted are the expression levels of these KSHV miRNAs in DG-75 cells (x-axis) and EA.hy926 relative to their BCBL1 levels.
(TIF)

Figure S3 Clustering of gene expression profiles follows first the cell line (DG-75 vs. EA.hy926), and within each cell line the treatment (transduction of KSHV miRNAs vs EGFP). Shown is the hierarchical clustering of all microarray samples on the Euclidean space of log2 expression levels with Ward linkage, and using all 15,678 genes monitored by the microarrays.
(TIF)

Figure S4 Correlation between changes in gene expression upon transducing the K10/12 vs EGFP constructs in DG-75 vs EA.hy926 cells for all 6916 genes whose expression is detectable in both cell lines.
(TIF)

Figure S5 mRNAs likely to be targeted by KSHV miRNAs are longer than mRNAs with no matches to KSHV miRNAs. The red and green histograms respectively represent the distribution of 3′ UTR length of the 1000 mRNAs with highest *KSHV miRNA sensitivity score* (see methods) and all mRNAs with no matches to KSHV miRNAs.
(TIF)

Figure S6 Caspase 3′UTR fragments are all potentially targeted by KSHV miRNAs. **A.** Schematic representation of Casp3 3′ UTR luciferase reporter and fragments. The seed-match types are described in the text. Either the full length 3′ UTR, or fragments spanning the UTR were cloned downstream of the firefly luciferase in the pSi-Check2 vector. **B.** Dual luciferase assays performed with the constructs depicted in **A**, no fragment of Casp3 UTR showed a stronger repression than the full-length UTR.
(TIF)

Figure S7 Tiny LNAs inhibition effect on KSHV miRNAs and Casp3 luciferase sensors. Dual luciferase assays performed with the indicated sensors co-transfected with the empty pcDNA or pcDNA expressing the K10/12 construct, and incubated with a mix of either control cel-miR-67, or with a mix of oligos antisense to miR-K12-1, -3, and 4-3p, at a final concentration of 1,5 µM. Luciferase ratios relative to empty psiCHECK-2 set to 1 are displayed.
(TIF)

Figure S8 Western blot analysis and signal quantification for PARP-1 and Tubulin on BC-3 cells treated with DMSO (left) or 0.5 µM Staurosporine for 8 h (right), and tiny LNA-oligonucleotides for control miR-67 (LNA-miR-67), or with a cocktail of oligonucleotides antisense to the seed region of miR-K12-1, K12-3, and K12-4-3p (LNA-miR-K12-1/3/4). Arrows and arrowheads indicate the signals corresponding to PARP-1, and cleaved PARP-1 respectively; the asterisk indicates a non-specific band. Though the juxtaposed lanes are not contiguous, all of them are from a single gel (indicated by the dotted line).
(TIF)

Table S1 Repartition of KSHV miRNAs in DG-75-K10/12 cells as assessed by small RNA cloning and Solexa-based sequencing.
(DOC)

Table S2 RT-PCR analysis of KSHV miRNAs expression in DG75 and EA.hy926 cell lines compared to BCBL1. n.d., not determined; mol., molecules
(DOC)

Table S3 Sequences of primers used in this study. Sequences of primers for luciferase miRNA sensors (A), target validation (B) and (C), miRNA expression in pcDNA (D), viral miRNAs qRT-PCR (E), cellular mRNAs qRT-PCR (F), tiny LNAs (G) and for mutagenesis of miRNA binding sites in Casp3 3′UTR (H) are all given 5′ to 3′.
(XLS)
