# Supplementary figures and images for: Kaposi's Sarcoma Herpesvirus microRNAs Target Caspase 3 and Regulate Apoptosis
Source: PLoS Pathog. 2011 Dec 8;7(12):e1002405. doi: 10.1371/journal.ppat.1002405 (PMC3234232; doi:10.1371/journal.ppat.1002405)

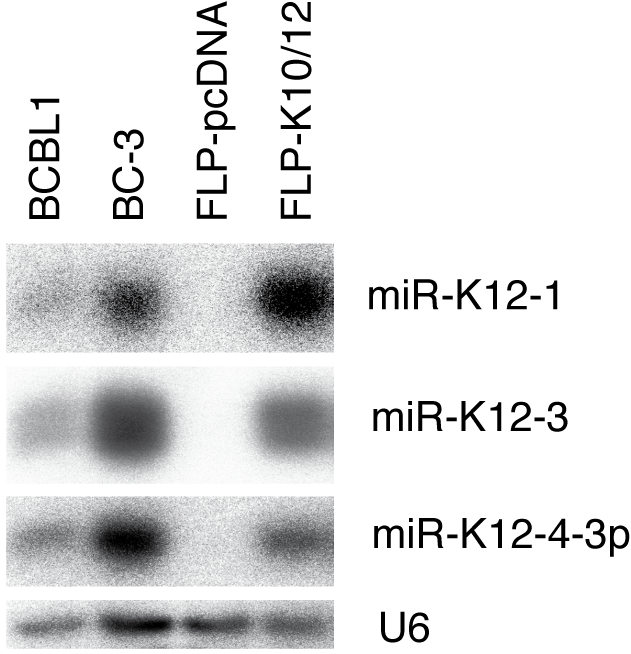

Supplement: Figure S1 — Northern blot analysis of BCBL-1, BC-3 and FLP-pcDNA and FLP-K10/12 cells grown in doxycycline-containing medium (final concentration of 1 µg/ml). U6 was used as a loading control. (TIF) [file ppat.1002405.s005.tif]

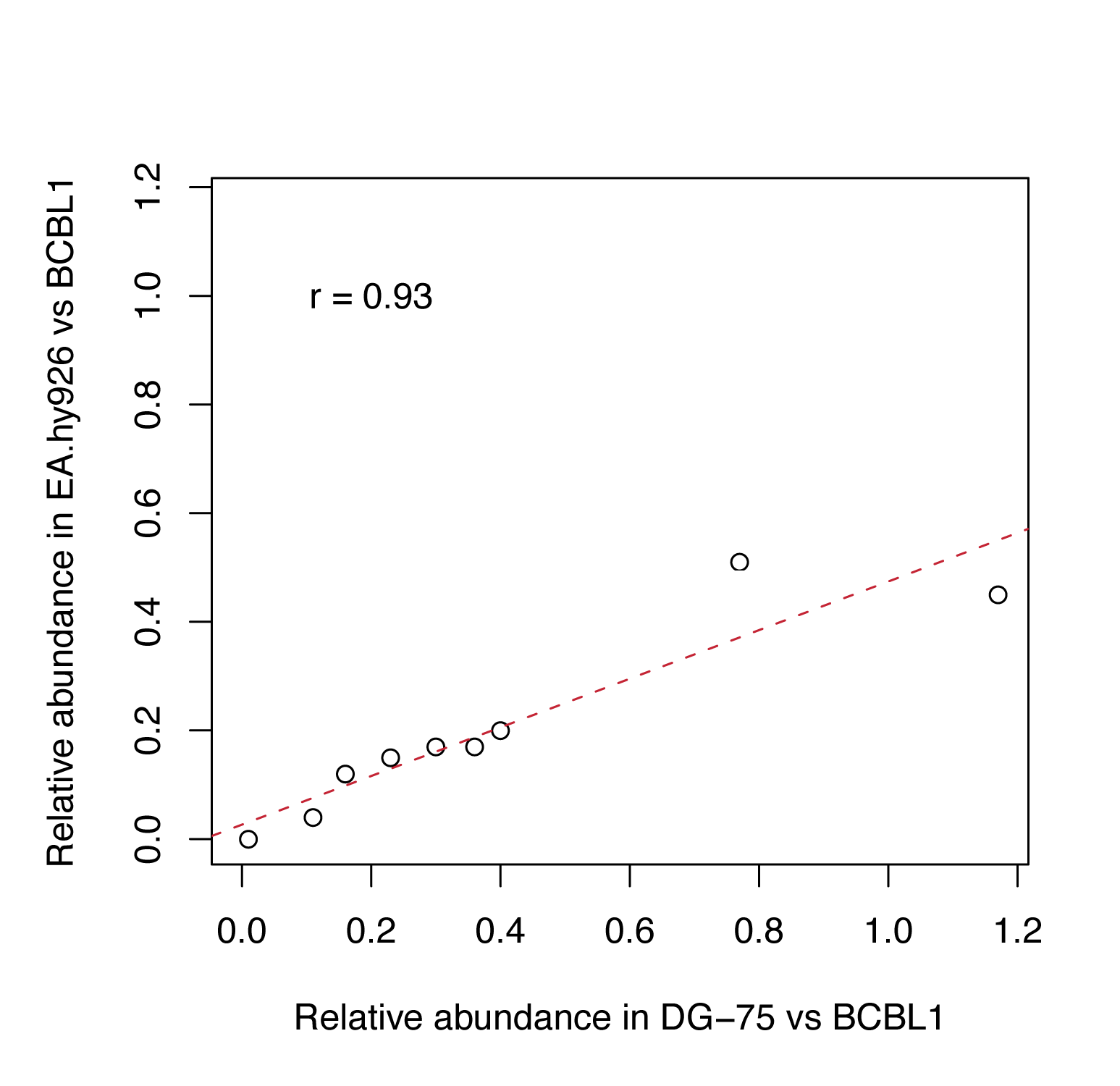

Supplement: Figure S2 — The relative abundance of KSHV miRNAs is similar in K10/12 transduced DG-75 cells and K10/12 EA.hy926 cells. Each dot on the scatter represents one KSHV miRNA whose expression in KSHV-infected BCBL1 cells, DG-75 cells and EA.hy926 was quantified by qPCR. Plotted are the expression levels of these KSHV miRNAs in DG-75 cells (x-axis) and EA.hy926 relative to their BCBL1 levels. (TIF) [file ppat.1002405.s006.tif]

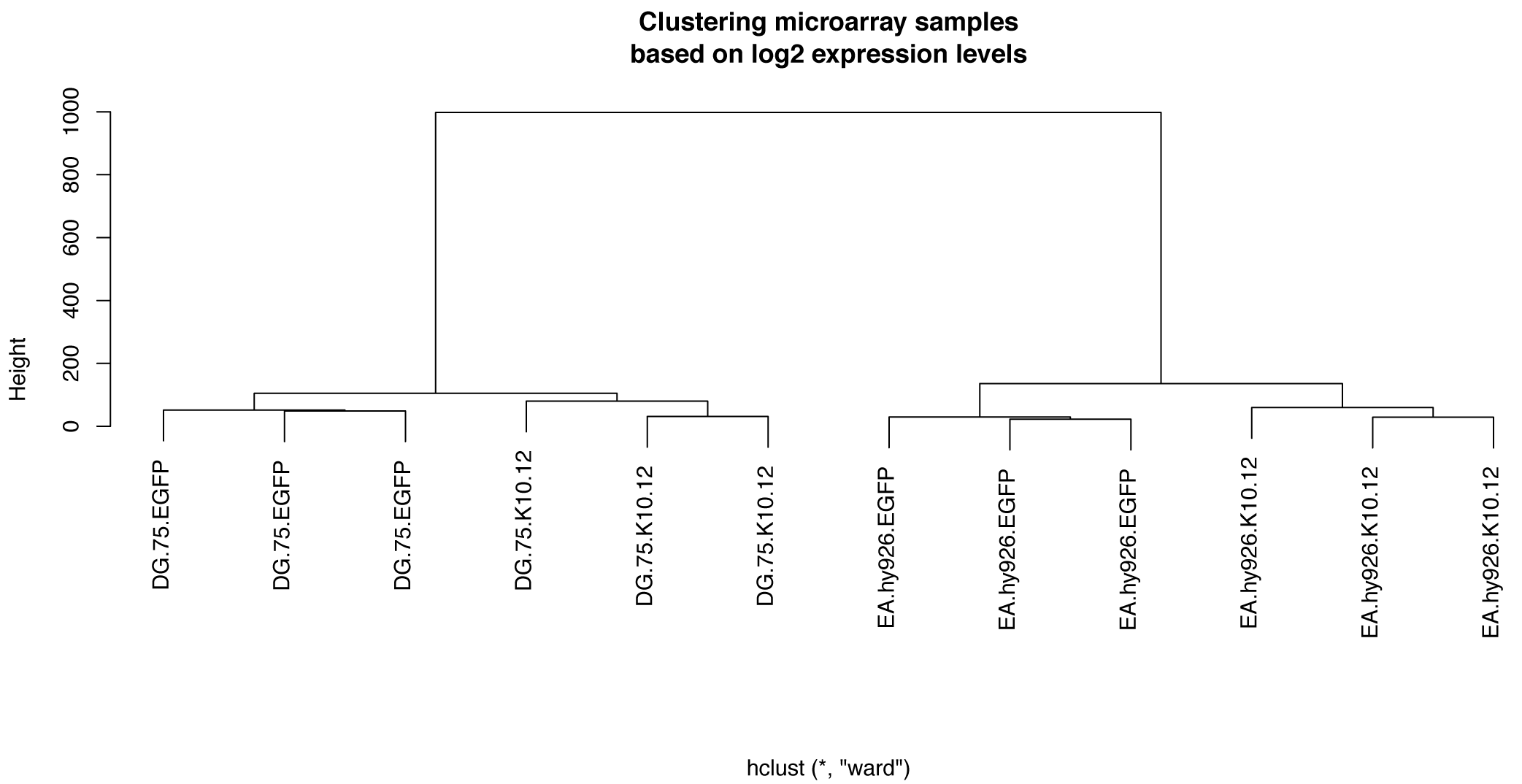

Supplement: Figure S3 — Clustering of gene expression profiles follows first the cell line (DG-75 vs. EA.hy926), and within each cell line the treatment (transduction of KSHV miRNAs vs EGFP). Shown is the hierarchical clustering of all microarray samples on the Euclidean space of log2 expression levels with Ward linkage, and using all 15,678 genes monitored by the microarrays. (TIF) [file ppat.1002405.s007.tif]

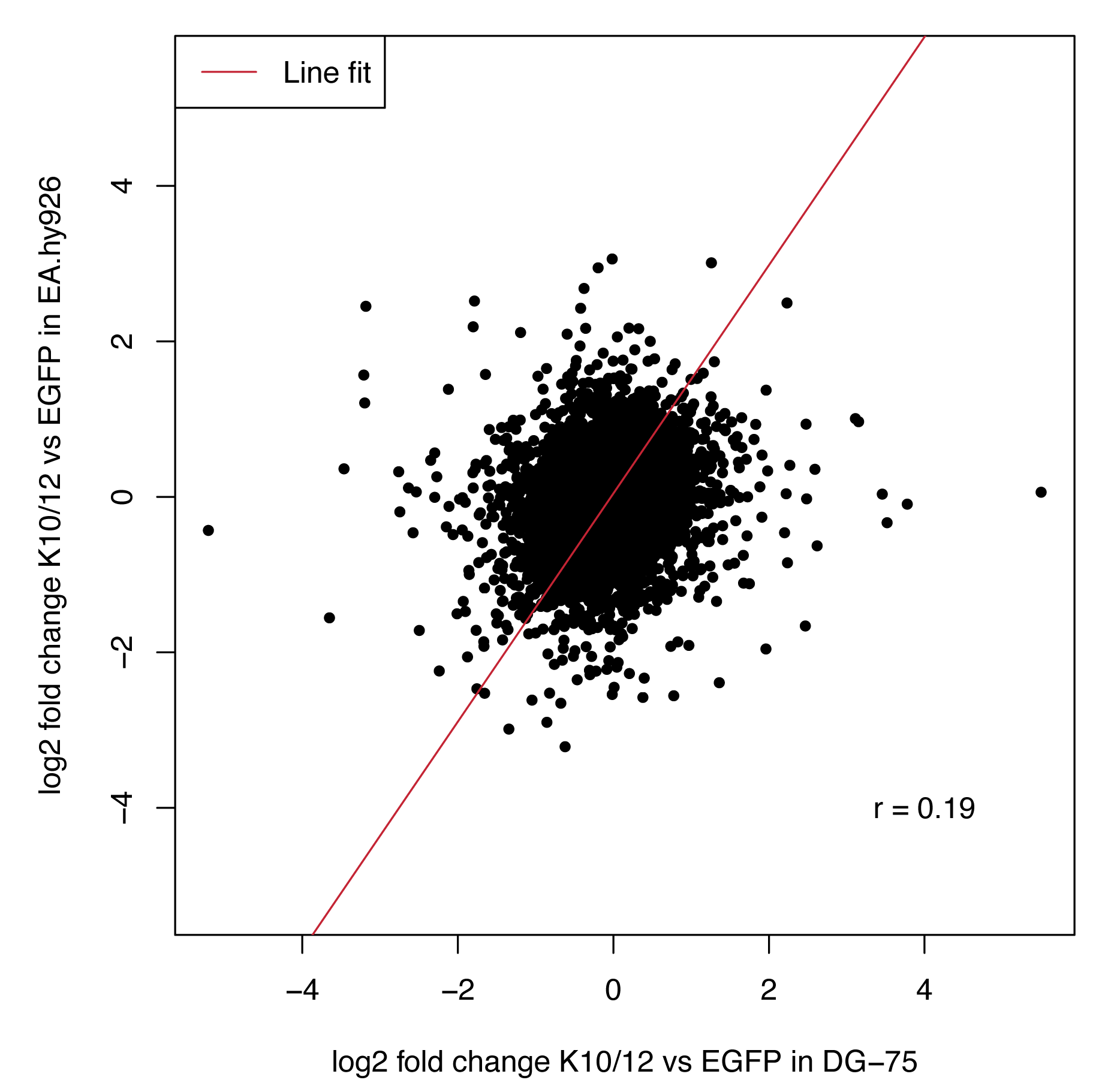

Supplement: Figure S4 — Correlation between changes in gene expression upon transducing the K10/12 vs EGFP constructs in DG-75 vs EA.hy926 cells for all 6916 genes whose expression is detectable in both cell lines. (TIF) [file ppat.1002405.s008.tif]

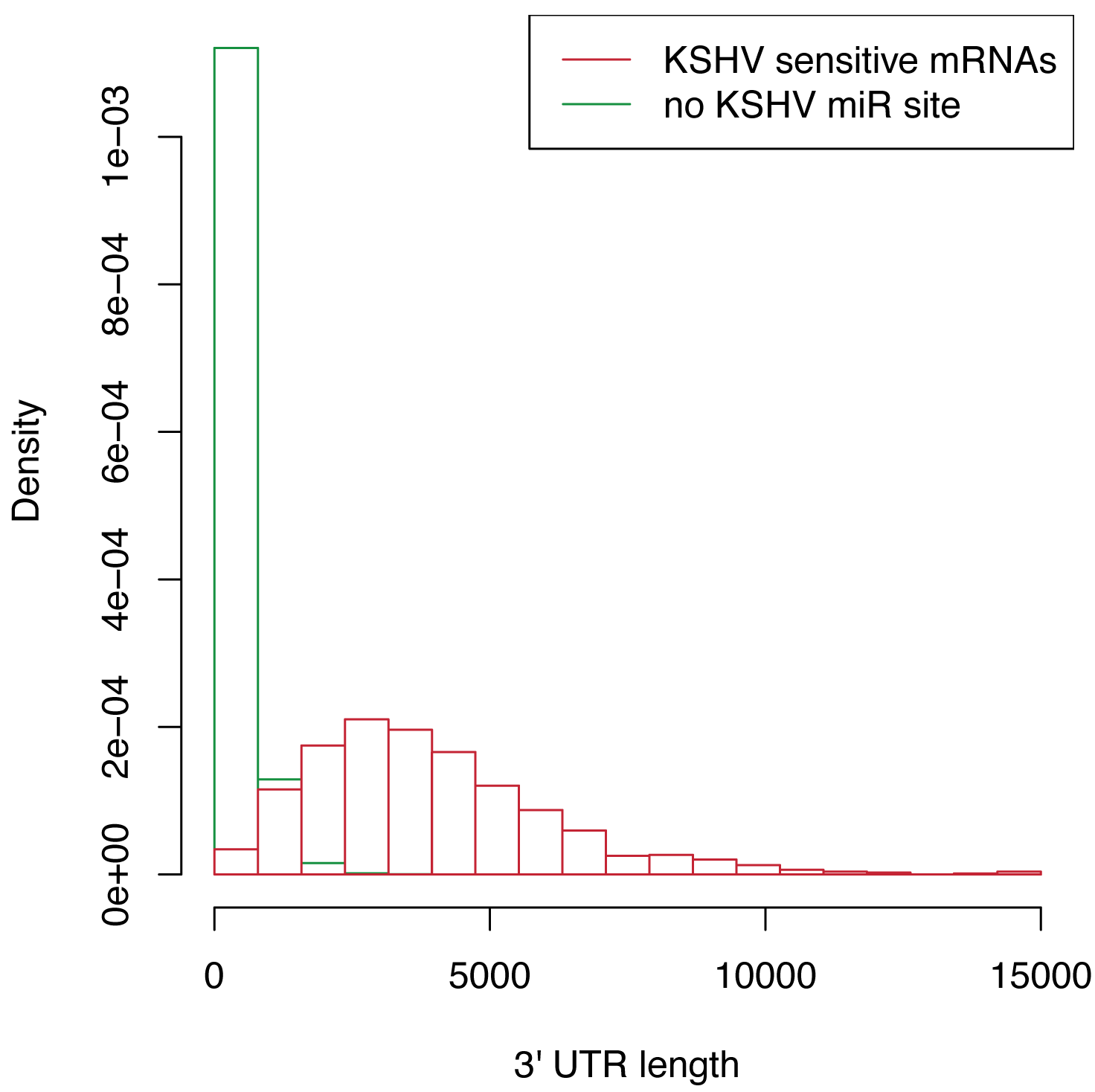

Supplement: Figure S5 — mRNAs likely to be targeted by KSHV miRNAs are longer than mRNAs with no matches to KSHV miRNAs. The red and green histograms respectively represent the distribution of 3′ UTR length of the 1000 mRNAs with highest KSHV miRNA sensitivity score (see methods) and all mRNAs with no matches to KSHV miRNAs. (TIF) [file ppat.1002405.s009.tif]

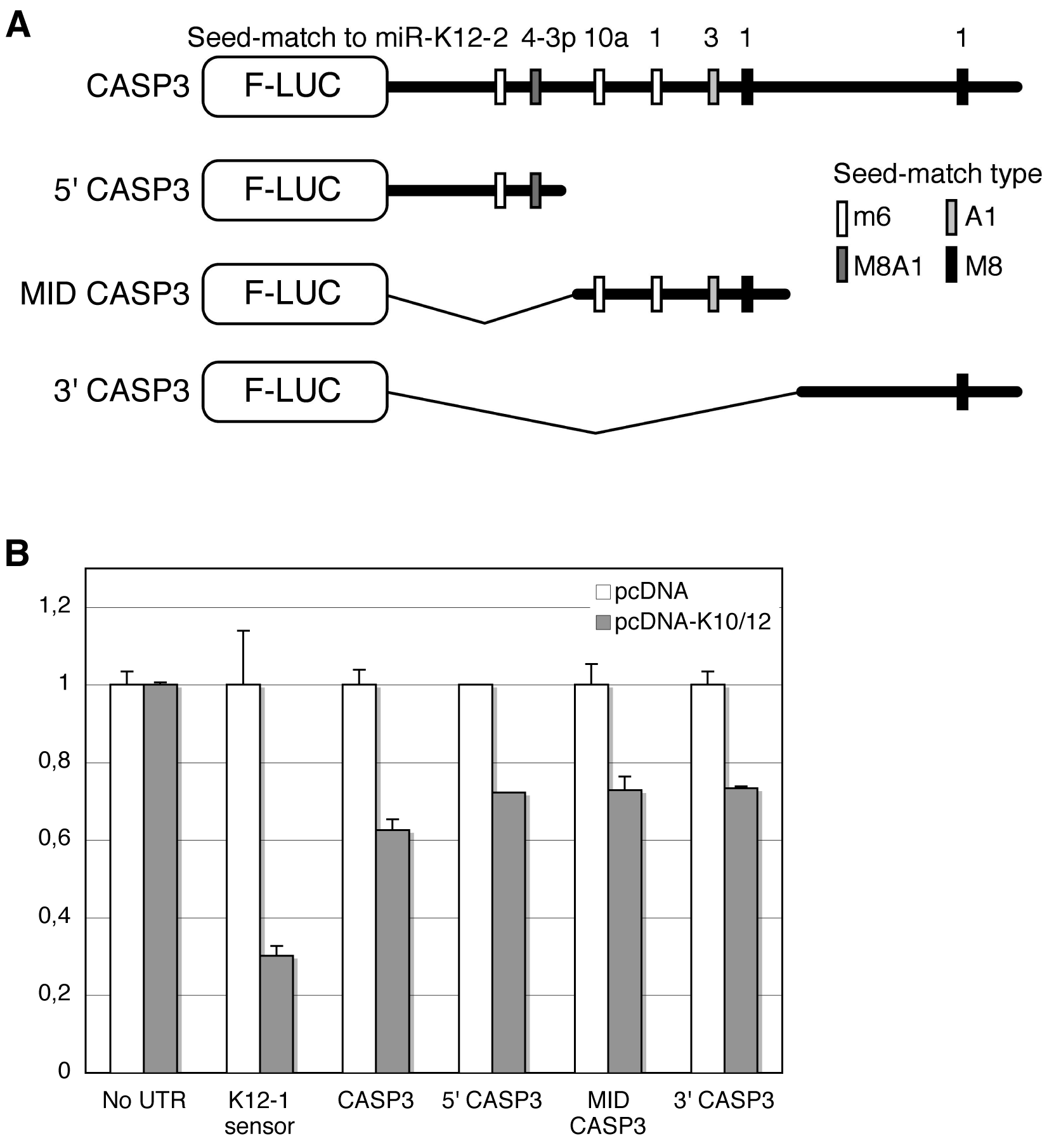

Supplement: Figure S6 — Caspase 3′UTR fragments are all potentially targeted by KSHV miRNAs. A. Schematic representation of Casp3 3′ UTR luciferase reporter and fragments. The seed-match types are described in the text. Either the full length 3′ UTR, or fragments spanning the UTR were cloned downstream of the firefly luciferase in the pSi-Check2 vector. B. Dual luciferase assays performed with the constructs depicted in A, no fragment of Casp3 UTR showed a stronger repression than the full-length UTR. (TIF) [file ppat.1002405.s010.tif]

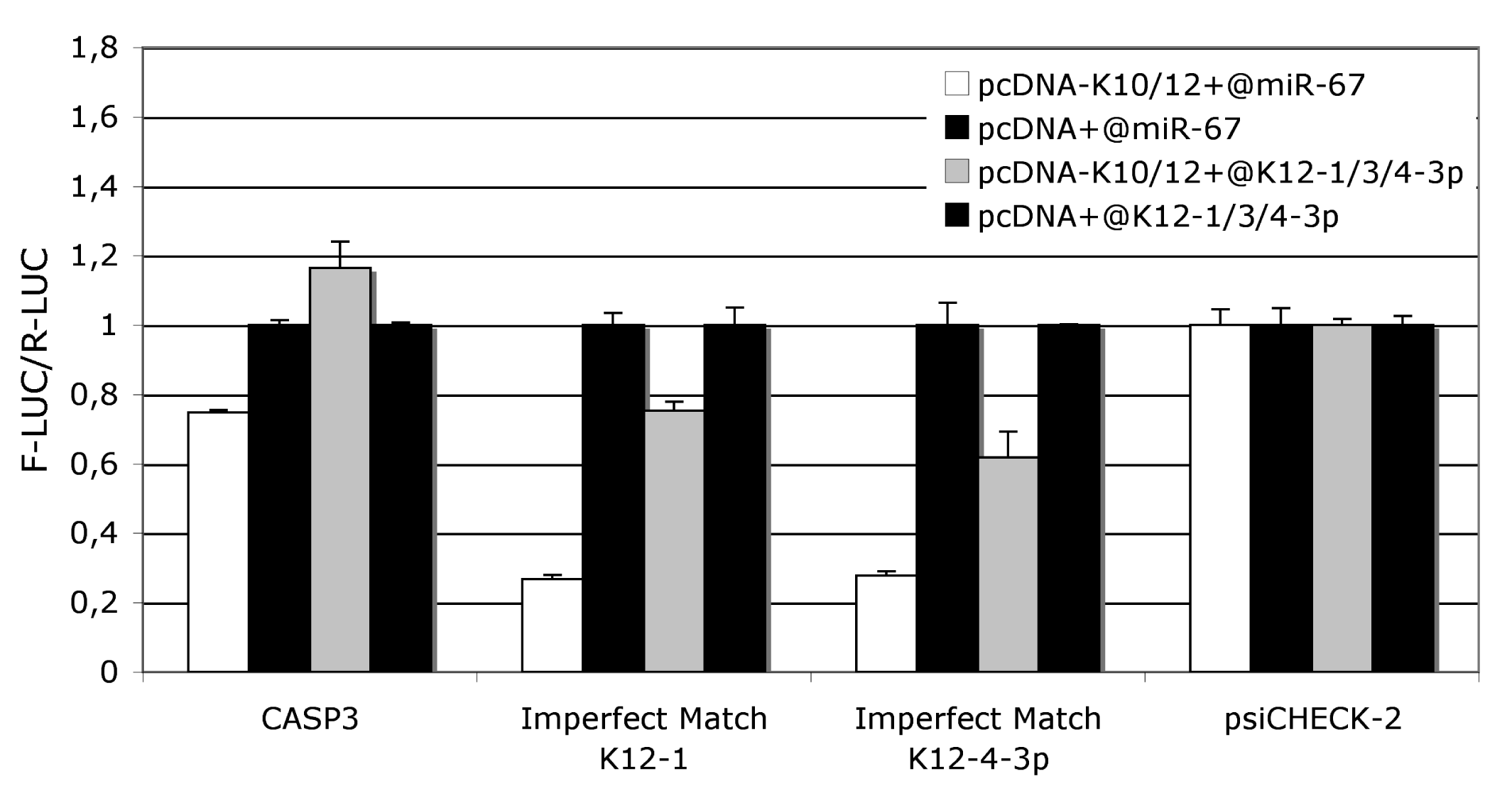

Supplement: Figure S7 — Tiny LNAs inhibition effect on KSHV miRNAs and Casp3 luciferase sensors. Dual luciferase assays performed with the indicated sensors co-transfected with the empty pcDNA or pcDNA expressing the K10/12 construct, and incubated with a mix of either control cel-miR-67, or with a mix of oligos antisense to miR-K12-1, -3, and 4-3p, at a final concentration of 1,5 µM. Luciferase ratios relative to empty psiCHECK-2 set to 1 are displayed. (TIF) [file ppat.1002405.s011.tif]

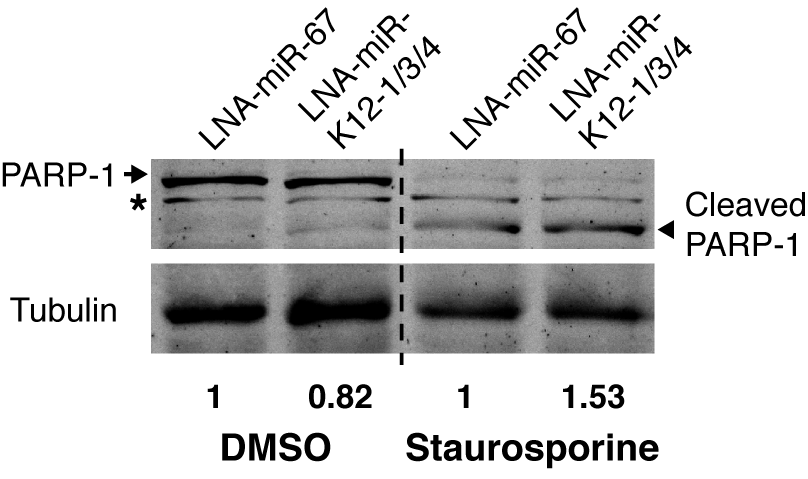

Supplement: Figure S8 — Western blot analysis and signal quantification for PARP-1 and Tubulin on BC-3 cells treated with DMSO (left) or 0.5 µM Staurosporine for 8 h (right), and tiny LNA-oligonucleotides for control miR-67 (LNA-miR-67), or with a cocktail of oligonucleotides antisense to the seed region of miR-K12-1, K12-3, and K12-4-3p (LNA-miR-K12-1/3/4). Arrows and arrowheads indicate the signals corresponding to PARP-1, and cleaved PARP-1 respectively; the asterisk indicates a non-specific band. Though the juxtaposed lanes are not contiguous, all of them are from a single gel (indicated by the dotted line). (TIF) [file ppat.1002405.s012.tif]
