# Supplementary material for: Kaposi's Sarcoma Herpesvirus microRNAs Target Caspase 3 and Regulate Apoptosis
Source: PLoS Pathog. 2011 Dec 8;7(12):e1002405. doi: 10.1371/journal.ppat.1002405 (PMC3234232; doi:10.1371/journal.ppat.1002405)
Supplement: Table S1 — Repartition of KSHV miRNAs in DG-75-K10/12 cells as assessed by small RNA cloning and Solexa-based sequencing. (DOC) [file ppat.1002405.s013.doc]

**Table S1**

|  |  | **DG75-K10/12** | |
| --- | --- | --- | --- |
| **miRNA** | **Sequence** | **Seq Number**  **(Total)** | **Seq Ratio**  **(per 100000)** |
| **miR-K12-1** | ATTACAGGAAACTGGGTGTAAGC | 192140 | 5555.31 |
| **miR-K12-1*** | GCAGCACCTGTTTCCTGCAACC | 107 | 3.09 |
| **miR-K12-2** | AACTGTAGTCCGGGTCGATCTGT | 65593 | 1896.48 |
| **miR-K12-2*** | GATCTTCCAGGGCTAGAGCTGC | 2471 | 71.44 |
| **miR-K12-3** | TCACATTCTGAGGACGGCAGCGA | 1483 | 42.88 |
| **miR-K12-3*** | TCGCGGTCACAGAATGTGACA | 24049 | 695.33 |
| **miR-K12-4-3p** | TAGAATACTGAGGCCTAGCTGA | 825458 | 23866.34 |
| **miR-K12-4-5p** | AGCTAAACCGCAGTACTCTAGG | 364425 | 10536.56 |
| **miR-K12-5** | TAGGATGCCTGGAACTTGCCGGT | 81900 | 2367.96 |
| **miR-K12-5*** | AGGTAGTCCCTAGTGCCCTAAG | 4 | 0.12 |
| **miR-K12-6-3p** | TGATGGTTTTCGGGCTGTTGAGC | 1191228 | 34441.79 |
| **miR-K12-6-5p** | CCAGCAGCACCTAATCCATCGG | 41652 | 1204.28 |
| **miR-K12-7** | TGATCCCATGTTGCTGGCGCTC | 4313 | 124.70 |
| **miR-K12-7*** | AGCGCCACCGGACGGGGATTTAT | 673 | 19.46 |
| **miR-K12-8** | CTAGGCGCGACTGAGAGAGCA | 642061 | 18563.81 |
| **miR-K12-8*** | ACTCCCTCACTAACGCCCCGCT | 884 | 25.56 |
| **miR-K12-9** | CTGGGTATACGCAGCTGCGTAA | 18352 | 530.61 |
| **miR-K12-9*** | ACCCAGCTGCGTAAACCCCGCT | 1820 | 52.62 |
| **miR-K12-11** | TTAATGCTTAGCCTGTGTCCGA | 57 | 1.65 |
| **KSHV miRNA** |  | 3458670 | 100000 |
| **Total miRNA** |  | 18498293 |  |
| **Ratio KSHV/Total (%)** | | 18.70 |  |
