# Supplementary material for: Kaposi's Sarcoma Herpesvirus microRNAs Target Caspase 3 and Regulate Apoptosis
Source: PLoS Pathog. 2011 Dec 8;7(12):e1002405. doi: 10.1371/journal.ppat.1002405 (PMC3234232; doi:10.1371/journal.ppat.1002405)
Supplement: Table S2 — RT-PCR analysis of KSHV miRNAs expression in DG75 and EA.hy926 cell lines compared to BCBL1. n.d., not determined; mol., molecules (DOC) [file ppat.1002405.s014.doc]

**Table S2**

|  | **DG75 K10/12**  (% of BCBL1) | **EA.hy K10/12**  (% of BCBL1) | **BCBL1**  (mol. per cell) |
| --- | --- | --- | --- |
| miR-K12-1 | 30% | 17% | n.d. |
| miR-K12-2 | 36% | 17% | 935 |
| miR-K12-3 | 77% | 51% | 51507 |
| miRK-12-4-5p | 1% | 0% | n.d. |
| miRK-12-4-3p | 11% | 4% | 9799 |
| miR-K12-5 | 23% | 15% | 126 |
| miR-K12-6-5p | 117% | 45% | 13312 |
| miR-K12-6-3p | 40% | 20% | 3543 |
| miR-K12-9-5p | 16% | 12% | n.d. |
